# Identification of breeding habitats and *kdr* mutations in *Anopheles* spp. in South Korea

**DOI:** 10.1186/s12936-023-04821-x

**Published:** 2023-12-16

**Authors:** Hyelee Hong, Tae-Hui Eom, Thuy-Tien Thi Trinh, Bao Duong Tuan, Hyun Park, Seon-Ju Yeo

**Affiliations:** 1 Department of Tropical Medicine and Parasitology, Department of Biomedical Sciences, College of Medicine, Seoul National University, Seoul, 03080 Republic of Korea; 2 Department of Tropical Medicine and Parasitology, Medical Research Center, Institute of Endemic Diseases, Seoul National University, Seoul, 03080 Republic of Korea; 3 Zoonosis Research Center, Department of Infection Biology, School of Medicine, Wonkwang University, 460 Iksan-Daero, Iksan, 54538 Republic of Korea

**Keywords:** *Anopheles sinensis*, *Anopheles belenrae*, Malaria, Breeding site, *Kdr* mutation

## Abstract

**Background:**

Malaria is still endemic in South Korea. However, limited information is available on the current *Anopheles* breeding sites and the occurrence of insecticide resistance-associated genetic mutations and their distribution needed to control the malaria vector efficiently.

**Methods:**

This study explored breeding sites of *Anopheline* adults in Gimpo-si, near the demilitarized zone (DMZ) in Gyeonggi-do province, South Korea, from 2022 to 2023. Genetic diversity was investigated based on the internal transcribed spacer (ITS2), cytochrome c oxidase subunit I (COI), and knockdown resistance (*kdr*) genes of *Anopheles* mosquitoes. A natural environment associated with the seasonal abundance of *Anopheles* larvae was characterized.

**Results:**

Two breeding sites of *Anopheles* larvae and adults were found at a stream margin or shallow freshwater near the forest in Wolgot-myeon in Gimpo-si without cattle shed within 1 km and in Naega-myeon in Ganghwa-gun with cow shed within 100 m in 2022 and 2023, respectively. Both sites were located between the newly cultivated lands and the forest. Besides, both breeding sites were in the valley at a slight elevation of 60–70 m from ground lands and maintained the shadow all day. Overall, the Wolgot-myeon breeding site showed various *Anopheles* spp. larvae, including *Anopheles sinensis*. Naega-myeon, an additional breeding site found in 2023, had *Anopheles sineroides* larvae, and approximately 59.7% (89/149) of *An. sinensis* adults inhabited within a 100-m distance. The total collection, including larvae and adults, revealed that *An. sinensis*, *Anopheles pullus*, *Anopheles kleini*, *An. sineroides*, *Anopheles belenrae*, and *Anopheles lindesayi* accounted for 44.2% (118/267), 0.7% (2/267), 0.7% (2/267), 22.1% (59/267), 1.9% (5/267), and 30.3% (81/267), respectively. Furthermore, various *kdr* mutant genotypes (F/F, C/C, L/F, L/C and F/C) in *An. sinensis*, and the first *kdr* allele mutant (L/F1014) in *An. belenrae* were identified in South Korea.

**Conclusions:**

Two breeding sites of *Anopheles* larvae were studied in Wolgot-myeon and Naega-myeon. Various *Anopheles* spp. larvae were detected in both habitats, but overall, *An. sinensis* was the most prevalent adults in both study sites. The occurrence of *kdr* allele mutant of *An. belenrae* in South Korea was reported. Rigorous larvae monitoring of *Anopheles* spp., continuously updating information on *Anopheles* breeding sites, and understanding the environmental conditions of *Anopheles* habitats are required to develop an effective malaria control programme in South Korea.

**Supplementary Information:**

The online version contains supplementary material available at 10.1186/s12936-023-04821-x.

## Background

Malaria is one of the major vector-borne diseases in South Korea, and *Anopheles* mosquitoes are a vector for transmitting *Plasmodium vivax* parasites [1]. A South Korean army soldier stationed near the Western edge of the demilitarized zone (DMZ) in northern Gyeonggi-do province was diagnosed with vivax malaria in July 1993. Since then, there has been an exponential increase in vivax malaria cases and epidemic outbreaks in South Korea, with 6,142 cases occurring up to 1998 [2, 3].

In South Korea, *Plasmodium falciparum* infection was present only among intravenous drug abusers, and indigenous falciparum malaria was not reported since 1945 [4]. *Plasmodium malariae* was also present before 1945, but has not occurred since [3]. Besides, political issues do not allow the movement of patients between South and North Korea. Thus, misdiagnosis or mixed infection with *P. falciparum* imported from abroad is not possible in South Korean residents with no history of overseas travel. *Plasmodium vivax* infects hepatocytes to form schizonts, causing blood infections. Dormant hypnozoites can persist for weeks to years after the primary infection before leading to blood infection relapse, making it difficult to estimate the incidence each year [5].

The South Korean epidemic area is adjacent to malaria-risk areas in North Korea, including the DMZ in Gyeonggi-do and Gangwon-do provinces. Although the exact size of the malaria epidemic in North Korea could be underestimated, approximately 5401–16,200 confirmed cases were reported in 2015 [6]. DMZ areas are highly preserved in the natural landscapes and have their ecosystems and biodiversity because no civilians have been allowed to enter the DMZ for over 70 years. Due to the isolated nature of DMZ, there is a potential for malarial cases and vectors in South Korea [3].

Mosquito-borne infections are closely related to climatic factors, such as temperature and precipitation. Optimum temperatures affect immature development, oviposition cycle, adult longevity, contact with hosts, and the extrinsic development of pathogens of medical and veterinary importance [7]. The larval development of mosquitoes is dependent upon water sources for species-specific habitats, e.g., tree holes/artificial containers, ponds, stream margins, and associated aquatic vegetation [8].

Previous studies suggested that the spatial distribution of malaria, transmitted by *Anopheles* mosquitoes, has shifted toward higher altitudes [8, 9]. There is a correlation between the relative abundance of mosquito populations and annual or monthly mean temperature and precipitation. Thus, continuous monitoring and habitat characterization of breeding sites of malarial vectors are urgently required.

Eight *Anopheles* species belonging to the Hyrcanus group *(Anopheles sinensis*,* Anopheles pullus*,* Anopheles belenrae*,* Anopheles kleini*,* Anopheles lesteri* and *Anopheles sineroides*), Barbirostris group (*Anopheles koreicus*), and Lindesayi complex are present in South Korea [1]. *Anopheles sinensis* is the most prevalent and important vivax malaria vector in South Korea [10]. However, *An. pullus, An. kleini, An. lesteri,* and *An. belenrae* have recently been shown to play an essential role in malaria transmission in the Gyeonggi Province of South Korea [11–13]. Especially, *An. pullus* and *An. belenrae* collected near DMZ were shipped to Thailand for analysis of the development of sporozoite. Upon feeding on the Thailand malaria patients infected with *P. vivax* parasites, salivary glands of two Korean mosquitoes were positive for *An. pullus* (3.4%; 6/175) and *An. belenrae* (5.1%; 7/137), indicating that *An. sinensis* and *An. belenrae* play a role in the transmission of malaria diseases in South Korea [14].

Informed larval interventions that target more prolific breeding sites have significant potential in combating *P. vivax* malaria, particularly at a regional scale [15]. The breeding habitat of *An. Sinensis* was reported to be widely present [10]. The larvae were found in rice fields, open grassy ponds, ground pools, swamps, marshes, shores of lakes, stream margins, ditches, and seepages in South Korea [16]. These are generally fresh, shallow water habitats, either stagnant or flowing, usually with emergent vegetation and exposed to sunlight [17]. However, limited information is available on the current breeding sites of larvae, with few reports in South Korea after 2007, although adult information is regularly reported in South Korea [1, 8, 18–20]. In contrast, China still reports the recent breeding sites of *Anopheles* larvae in rice paddies, streams, ponds, and rice fields [15, 21].

Public health pesticides have been used extensively to reduce field vectors, and insecticides have been widely used to control arthropod pests, including mosquitoes [22]*.* In rural South Korea, pesticides, such as mosquito repellants and agricultural chemicals known to contain fast-acting pyrethroids as an active pesticide ingredient, are commonly used [23]. The South Korean Military National Defense increased the use of pesticides, such as Deltamethrin and DDT, for vector control from 2011 to 2013 [19], causing resistance to pyrethroids and insecticides in *An. sinensis* [23, 24]. Currently, pyrethroids are still the most common pesticides used in South Korea [25].

Knockdown resistance (*kdr*) is a well-characterized mechanism of resistance to pyrethroids and insecticides in many insect species associated with a mutation in codon 1014 of the voltage-gated sodium channel (VGSC) gene [26]. Therefore, *kdr* genotyping has been included in monitoring pyrethroid resistance in *An. sinensis* in South Korea, while other countries have reported the *kdr* mutation in other *Anopheles* species [27, 28]. The insecticide resistance status in other *An. sinensis* species is not adequately understood in South Korea. Based on the historical intensive use of insecticides to control malaria in South Korea, it is necessary to monitor the occurrence of resistance to pyrethroids and insecticides that may contribute to its management in other *An. sinensis* species. This study investigated current *Anopheles* breeding sites and identified *kdr* mutations impacting pyrethroid resistance in field populations of *Anopheles* mosquitoes collected across Incheon and Gyeonggi-do province near the DMZ.

## Methods

### Study area

Larvae and adults of *Anopheles* mosquitoes were collected from Wolgot-myeon (37$^\circ$ 44′ 42″ N, 126$^\circ$ 32′ 04″ E) in Gimpo-si, Gyeonggi-do and Naega-myeon (37$^\circ$ 43′ 52″ N, 126$^\circ$ 24′ 55″ E) in Ganghwa-gun, Incheon, South Korea during July to early October in 2022 and 2023.

### Larvae collections

Standard dippers with approximately 500 mL volume were used to collect larvae from the water bodies, as previously described [29]. Ten dips of water were taken to determine the presence of larvae. All species of larvae were morphologically identified using the microscope guidelines in the field and brought to the laboratory for molecular diagnosis [29].

### Adult mosquito collections

Adults were captured in Blackhole Mosquito Buster traps (BioTrap, Seoul, Korea), which were set up near the villages, and mosquitoes were captured from 18:00 to 09:00. The captured mosquitoes were transported to the laboratory within 2 h and morphologically identified as species or *An*. *hyrcanus* group using a dissecting microscope and standard keys [30].

### Preparation of genomic DNA

The mosquitoes were stored at − 20 °C. For the molecular identification of the *Anopheles* species and the presence of *P. vivax*, the mosquitoes were separated at the head, including the salivary gland, midgut, and wings. From each head and midgut, genomic DNA was extracted by Chelex (Bio-Rad, Hercules, CA, USA) [31]. Each part, the head, and the midgut were grounded with 20 μL of distilled water (DW). Subsequently, 100 μL autoclaved phosphate-buffered saline (PBS) was added, gently vortexed, and incubated at room temperature for 20 min, followed by centrifugation at 20,000*g* for 2 min. The supernatant was discarded, and the pellet was washed with 100 μL of PBS, dissolved in 75 μL of Tris–EDTA (TE) buffer and 25 μL of 20% w/v Chelex-100 resin suspension. Subsequently, the samples were boiled for 10 min, followed by centrifugation at 20,000*g*, and the supernatants were used as template DNAs.

### Polymerase chain reaction (PCR)

Universal primers for the internal transcribed spacer (ITS2) gene of *Anopheles* adult were used as previously reported: (An-ITS2-U1; forward primer, 5ʹ-ATCGATGAAGACCGCAGCTA-3′/reverse primer, 5′-CAACACGACTCCATGGTACG-3′) [32]. In addition, the cytochrome c oxidase subunit I (COI) gene was amplified by PCR using the following primers as previously described: (forward primer, 5ʹ-GGATTTGGAAATTGATTAGTTCCTT-3ʹ/reverse primer, 5ʹ-AAAAATTTTAATTCCAGTTGGAACAGC-3ʹ [33]. For identification of the knockdown resistance (*kdr*) gene, primers were chosen as previously described (5′ASIIS56; forward primer, 5′-CGGACTTCATGCACTCCTTCA-3′/reverse primer, 5′-TTAGCGCATTTGCTA CGTTC-3′) [24].

PCR was conducted in a 25 μL reaction mixture containing 0.4 μM of each primer, 1xPCR buffer, 0.2 mM of each deoxynucleoside triphosphate (dNTP), 0.5 units Taq DNA polymerase (Takara Bio, Inc., Otsu, Japan), and 100 ng of genomic DNA.

PCR amplification was performed under conditions of denaturation at 94 °C for 5 min, 35 cycles of denaturation at 94 °C for 30 s, annealing at 55 °C for 30 s, extension at 72 °C for 2 min, and final extension at 72 °C for 5 min with a PCR machine (Bio-Rad). After confirmation of the bands, the PCR products were sent for sequencing (Cosmogenetech, Seoul, South Korea). Multiplex PCR was conducted with universal forward and species-specific reverse primers designed for eight species of *Anopheles* present in South Korea, as previously reported [32]. Briefly, An-ITS2-U1 was used as the universal forward primer (5′-ATCGATGAAGACCGCAGCTA-3′), and distinct reverse primers were used for *An. sinensis* (5′-TAGGGTCAAGGCATACAGAAGG-3′ (1112 bp), *An. koreicus *(5′-TATCGTGGCCCTCGACAG-3′ (925 bp), *An. lindesayi,* 5′-ACCATCTACTGCCTGAACGTG-3′ (650 bp), *An. kleini,* 5′-TTTGTTGATAACTTGTATCGTCCATC-3′ (527 bp), *An, lesteri,* 5′-CAGTCTCTTGCAGCCCATTC-3′ (436 bp), *An. sineroides,* 5′-CGCGCACGCTCAGATATT-3′ (315 bp), *An. belenrae,* 5′-TGTCCTAGGCGGTTATCAACA-3′ (260 bp), and *An. pullus,* 5′-CGGCGTAGTTTATTGTGTATAACATC-3′ (157 bp).

### Real-time PCR

To determine the RNA copy number of *P. vivax*, plasmids containing the plasmodial 18S rRNA gene were synthesized as a positive control DNA, using Cosmogenetech (Daejeon, South Korea) as previously reported [34]. For the detection of *P. vivax*, 159-bp segment of the four plasmodial 18S genes was amplified using real-time PCR with Plasmo 1 as a forward primer (5′-GTTAAGGGAGTGAAGACGA TCAGA-3′) and Plasmo 2 as a reverse primer (5′-AACCCAAAGACTTTGATTTC TCATAA-3′). *Plasmodium vivax*-specific probe (5′-HEX-AGCAATCTAA GAATAAACTC CGAAGAGAAA ATTCT-TAMRA-3′) was able to differentiate it from other *Plasmodium* parasites [34].

The real-time PCR was conducted in a 20 μL reaction mixture containing 0.5 μM each primer Plasmo 1 and 2, 0.5 pM probe, 1xQuantiTect Probe RT-PCR Master Mix, 0.2 μL of QuantiTect RT Mix (QIAGEN, Hilden, Germany), and 10 ng of genomic DNA extract of mosquitoes. PCR amplification was performed with denaturation for 15 min at 94 °C, followed by 45 cycles of 30 s at 94 °C, 1 min at 60 °C.

### Phylogenetic tree

Multiple ITS2 sequences and COI of *Anopheles* species available in the GenBank library were selected using the Basic Local Alignment Search Tool (https://blast.ncbi.nlm.nih.gov/Blast.cgi). Phylogenetic trees were created using a maximum-likelihood method, and the tree topology was set for 1000 replications (bootstrap) to determine the statistical significance using MEGAX version 10.2.6 (https://www.megasoftware.net/).

### Breeding of larvae and adults

The captured female adults near a cow shed were moved to 20 oz cups filled with water to lay eggs. After laying eggs, the female was used to identify species by PCR-targeted ITS2 gene. The identified *An. sinensis* was used in this study. The eggs were collected in 200 mL 0.02% yeast slurry to hatch. Larvae were fed the ground TetraMin fish food flakes (Tetramin Tropical Flakes-Spectrum Brands, Blacksburg, VA, USA) and vitamins as the culture media. The adults were moved to a cage and kept at 60 ± 10% humidity while feeding 10% sucrose soaked in cotton balls.

### Insecticide preparation

Etofenprox powder was purchased from Sigma-Aldrich (St. Louis, WA, USA). Etofenprox stock solution was prepared by diluting the active ingredient in acetone and stored in Eppendorf tubes covered in aluminum foil to prevent UV exposure. The lid was wrapped in parafilm to reduce evaporation.

### Adult topical bioassay

The reared adult females *An. sinensis* mosquitoes were tested using a topical application described by the World Health Organization [35]. Mosquitoes (unfed 1–5 days old) were capped out of a cage into Eppendorf tubes placed on ice. Mosquitoes remained on ice for at least 5 min before being topically treated with the pyrethroid insecticide or solvent control. Based on the reference LD_50_ concentration [36], 2.41 μg/♀, mosquitoes were treated 10 times low and 100 times high doses, including the reference concentration. Anaesthetized mosquitoes were treated topically with 0.1 μL of a given concentration of each insecticide dissolved in the reagent-grade solvent. Reagent-grade acetone alone was used as the negative control. Treatments were applied to the pronotum of each test mosquito. Treated insects were placed in respective insect breeding boxes (SPL, Seoul, South Korea) with 10% sucrose on the top of the mesh lids and held at a constant room temperature at 24 ± 2 °C and 60 ± 10% RH. The number of dead or moribund insects was counted 24 h after treatment. The insects were considered dead if they did not move when plastic cups were impacted. All treatments were replicated three times using 4 or 5 adult females per replicate.

### Larval bioassay

A direct-contact mortality bioassay [37] was used to evaluate the toxicity of etofenprox to third-instar larvae from reared *An. sinensis* populations. Groups of 10 mosquito larvae in the insect breeding box (SPL, Seoul, South Korea) were separately exposed to each concentration (250 mL). The toxicity of insecticide was determined with four to six concentrations ranging from 0.65 to 54.8 mg/L in serially diluted culture media. Negative controls consisted of acetone in culture media. After 24 h post-treatment, a larva was considered dead if it did not move when sucked with a disposable spoide. Freshly prepared culture media were used for bioassays.

### Visualization of contour line and shadow

The National Geologic Map Database (https://ngmdb.usgs.gov/ngmdb/ngmdb_home.html) was used to visualize the contour line. The ground area surrounded by valleys, including breeding sites, was calculated by circumference line using Google Earth tools.

ShaowClauculaor (http://shadowcalculator.eu) was used to depict the shadow direction. It displayed mountain shadows on Google Maps for two breeding locations at each time.

### Statistics

Lethal dose (LD_50_), R-squared, and 95% confidence intervals (CI) were calculated via nonlinear regression followed by interpolation of log(inhibitor) vs. normalized response—Variable slope graphs using GraphPad Prism 9.0. Results were presented as the mean ± SD. Statistical significance was set at *P* < 0.05.

## Results

### Characteristics of potential breeding sites for *Anopheles*

**Fig. 1:**
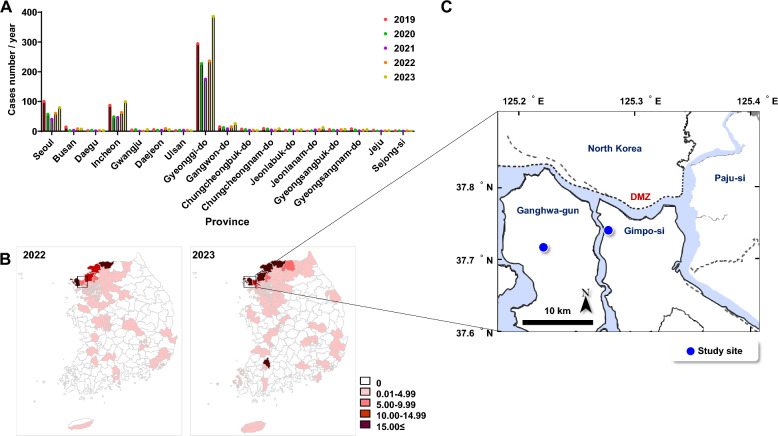
Incidence of malaria cases and study places near DMZ in South Korea. **A** Recent malaria cases in each province of South Korea during the last 5 years. **B** Map shows the geographical distribution of the malaria incidence rate per 100,000 population in South Korea in 2022 and 2023. Insert is the study sites. **C** Two breeding sites (blue circles) in Wolgot-myeon in Gimpo-si and Naega-myeon in Ganghwa-gun with the highest malaria incidence were explored

Malaria is prevalent in South Korea, with an average of 500 cases annually. COVID-19 disrupted malaria services, leading to a significant increase in cases and deaths worldwide [38]. Interestingly, the number of malaria cases in Korea decreased below 274 during the COVID-19 pandemic, rising to more than 420 and 649 cases in 2022 and 2023, according to the South Korea Disease Control and Prevention Agency [39]. Gyeonggi-do province near the DMZ had the highest malaria incidence, significantly increasing yearly (Fig. 1A). The study was first carried out in five villages in Western South Korea in 2022 (Additional file 1: Figure S1). Among those areas, Wolgot-myeon was a positive site as a breeding habitat in 2022, and an additional site Naega-myeon was found in Ganghwa-gun in 2023. The incidence rate increased from 7. 21 to 15.45 in Wolgot-myeon in Gimpo-si and from 17. 2 to 18.64 in Naega-myeon in Ganghwa-gun per 100,000 population during 2022 and 2023 (Fig. 1B). Interestingly, the incidence rate increased from 2.17 to 21.75 in Jeonranam-do in the Southern province during 2023. Wolgot-myeon is located between 37° 44′ 42″ N and 126° 32′ 04″ E (altitude 61 m), and Naega-myeon is between 37° 43′ 52″ N and 126° 24′ 56″ E (altitude 71 m). Figure 1C displays two study sites (Wolgot-myeon in Gimpo-si; Naega-myeon in Ganghwa-gun) on Google Maps, showing adults and larvae of *Anopheles* mosquitoes.

### Differential distribution of mosquito larvae and adults in freshwater swamp forest

Previous studies have demonstrated that *An. sinensis* larvae were commonly found in ground pools, plastic containers, swamps, and rice fields in South Korea [15, 40]. Gimpo-si was reported to have a high abundance of *An. sinensis* larvae [41]. The rice paddies in Gimpo-si from May 2022 were searched because the abundance of *Anopheles* larvae in DMZ areas was not well reported. In the DMZ areas, the presence of seven species (*An. belenrae, An. kleini, An. lesteri, An. lindesayi, An. pullus, An. sinensis,* and *An. sineroides*) has been reported [41]. *Anopheles sinensis* and *An. lesteri* populations peaked in August, whereas *An. pullus* peaked in May, decreasing from June to October in South Korea [16].

The habitat type was identified, and the surface area of the breeding site was categorized into one of three groups: (i) small habitat area (< 1 m^2^); (ii) medium habitat area (1–10 m^2^); and (iii) large habitat area (> 10 m^2^) as previously described [42]. Two breeding sites were found in this study in Gimpo-si and Ganghwa-gun near DMZ. Following previous guidelines, up to 10 dips were taken with a standard 350 mL dipper from every breeding site [43]. The *Anopheles* larvae in the breeding sites were lying flat on the water surface to breathe air through spiracular openings (Additional file 1: Figure S2).

The presence of larvae at high or low densities was categorized by the dipping method as previously described [43]. If *Anopheles* could be seen without dipping or nearly every dip contained *Anopheles* larvae, the site was defined as having a high *Anopheles* density. Sites where only one or two dips out of 10 contained *Anopheles* larvae were recorded as having a low density. Sites where no *Anopheles* larvae could be found in ten dips were defined as empty.

**Fig. 2:**
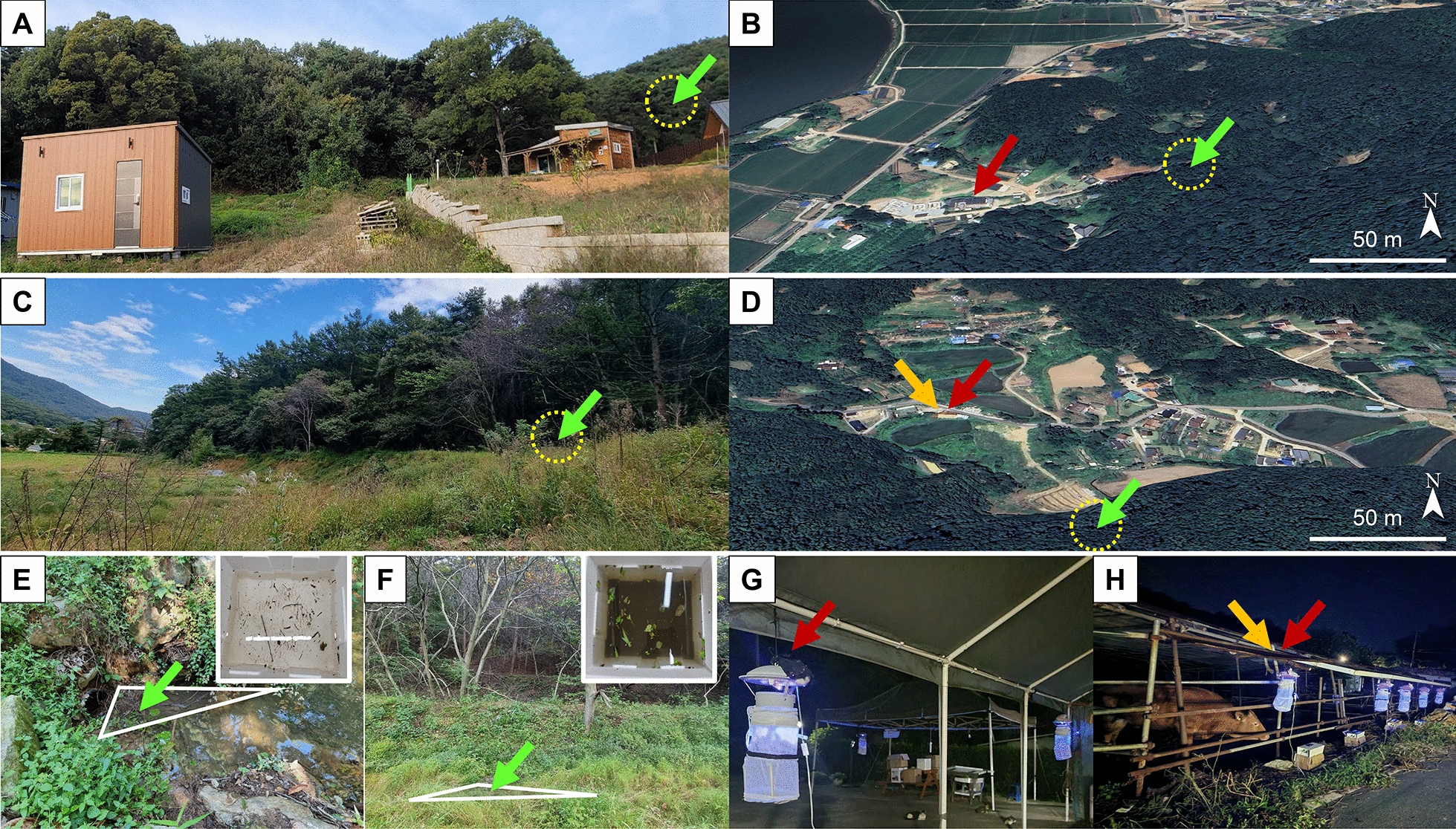
Study sites of *Anopheles* spp. larvae and adults in Gimpo-si and Gangwha-gun, 2022–2023. **A**, **B** Wolgot-myeon in Gimpo-si was investigated for mosquito larvae and adults in 2022–2023. **C**, **D** Naega-myeon in Ganghwa-gun was investigated for mosquito larvae and adults in 2023. Surface area and turbidity (insert) of breeding habitats were measured in Wolgot-myeon (**E**) and Naega-myeon (**F**). Green -, orange-, and red arrows indicate the sites of breeding, cattle shed for trapping adults, and human dwelling for trapping adults, respectively. White-lined triangle zone is the place of the presence of larvae. Traps were installed in human dwellings in Wolgot-myeon and **G** cattle sheds in Naega-myeon (**H**) within 100 m distance of breeding sites

The *Anopheles* larvae found at 2–5 larvae /dipping near the edge of the stream water in front of a forest of Wolgot-myeon in Gimpo-si (Fig. 2A and B), and the wet meadow with a freshwater marsh in front of a forest of Naega-myeon in Ganghwa-gun in 2023 (Fig. 2C and D) were considered as low abundance. The stream was placed behind a newly constructed house dwelling in front of the forest in Wolgot-myeon, and newly cultivated lands were developed in front of the breeding site within 50 m radius in Naega-myeon (Fig. 2A and C). Google Map shows the distance between the trap/cattle shed and breeding sites, indicating that the breeding sites were located within less than a 100 m radius (Fig. 2B and D).

The breeding spot of Wolgot-myeon was present by a cul-de-sac oMn the street connected with the forest entrance. At the corner of the cul-de-sac, a freshwater stream of forest (water surface area of the breeding site; 2 m^2^) was located and connected with the upper stream of the forest (Fig. 2E). Besides, a meadow area in front of a forest in Naega-myeon of Ganghwa-gun was found to breed *Anopheles* spp. larvae in shallow freshwater (Fig. 2F). The turbidity of the breeding habitats was classified into one of the following categories: (i) transparent; (ii) turbid, when the bottom of the dipper was still visible; or (iii) very turbid, when the bottom of the dipper was invisible as previously reported [42]. By using the dipper, the turbidity of Wolgot-myeon stream water was determined to be transparent as the dipper was visible (insert of Fig. 2E), but Naega-myeon showed a very turbid level (insert of Fig. 2F), implicating that the habitats might possess different species among larvae population.

For each study, five traps were installed at dwelling houses in Wolgot-myeon within 100 m from the breeding site at 20:00 to 09:00 from August to September 2022 (Fig. 2G) and in the cattle shed in Naega-myeon at 20:00 to 09:00 in August to September 2023 (Fig. 2H).

### Distribution of mosquitoes in the study sites

The larval sampling was conducted using the standard dipping method at 10 times dipping with a plastic container (30 cm × 50 cm × 20 cm), as previously described [15].

Table 1 shows the total mosquito collection from July to October, indicating that despite a permanent breeding site within a 100 m radius, Wolgot-myeon was in the low abundance *Anopheles* spp. adult category. Since collection of larvae at the breeding site was started in Wolgot-myeon on August 24th, 2022, the number was not indicative of the entire August. After collection, larvae were transported and reared in the laboratory to make adults. Thus, the adult population in 2022 included captured adults and those developed from larvae.

**Table 1:** Number of *Anopheles* spp. collected from different places in South Korea, 2022–2023

Place	Stage	2022				2023			
		Jul	Aug	Sep	Oct	Jul	Aug	Sep	Oct
Wolgot−myeon	Larva	ND^b^	100	300	20	10	30	70	40^c^
	Adult^a^	ND	24	7	2	20	6	ND	ND
# of larva and adult		ND	124	307	22	30	36	70	40
# of identification (%)^d^		ND	17.7 (22/124)	4.9 (15/307)	40.9 (9/22)	60.0 (18/30)	8.3 (3/36)	72.9 (51/70)	ND
Naega-myeon	Larva	ND	ND	ND	ND	ND	5	90	180^e^
	Adult	ND	ND	ND	ND	26	190	99	ND
# of larva and adult		ND	ND	ND	ND	26	195	189	180
# of identification (%)		ND	ND	ND	ND	ND	17.9 (35/195)	31.2 (59/189)	30.6 (55/180)
# of total larva and adult		ND	124	307	22	56	231	259	220
# of total identification (%)		ND	17.7 (22/124)	4.9 (15/307)	40.9 (9/22)	32.1 (18/56)	16.5 (38/231)	42.5 (110/259)	25.0 (55/220)

^a^Captured or developed from field larvae; PCR-positive samples were analyzed for further work

^b^Not done

^c^Collected within the first week of October, and most were in the first larvae stage

^d^# of species identified by PCR / # of total collection

^e^Collected for four consecutive days, maintained in the third or fourth larva stage and none in the first or second larvae stage

Since Wolgot-myeon had a low abundance of adults in 2022, alternative breeding sites were searched in 2023. Adults at a cattle shed were found in Naega-myeon in July 2023 but could not find a breeding habitat near the cattle shed in July. Finally, in August a breeding spot was detected with five larvae in the fresh shallow water of a meadow.

While a human dwelling in Wolgot-myeon maintained a low number of adults (July, *n* = 20; August, *n* = 6) in 2023, cattle shed in Naega-myeon showed an increased number of blood-fed adults in July (*n* = 26), approaching the peak in August (*n* = 190), and the number decreased in September (*n* = 99).

Interestingly, the number of larvae in Wolgot-myeon was decreased in 2023 compared with 2022, but the breeding site in Naega-myeon still had > 90 larvae in September, maintaining high numbers until the first week of October 2023. Besides, the larvae in Wolgot-myeon stayed at the stream edge under the shadow of trees, and the Naega-myeon breeding site in the shallow freshwater was covered by meadow grass, keeping humidity efficiently during September and October.

**Fig. 3:**
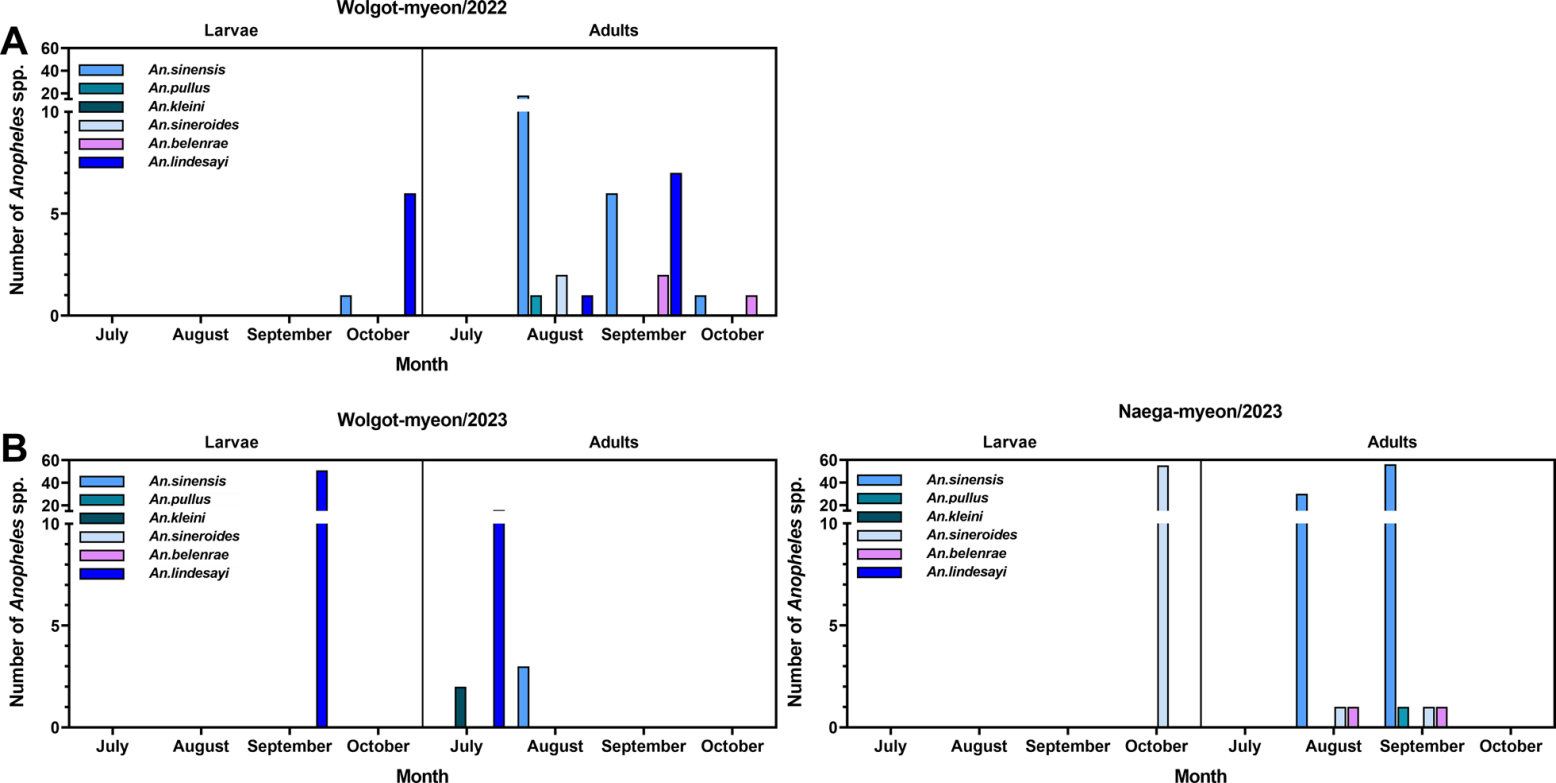
Number of *Anopheles* spp. identified at two breeding sites. **A** Wolgot-myeon in 2022, **B** Wolgot-myeon and Naega-myeon in 2023

Overall, *An. sinensis* was the most prevalent species among *Anopheles* in both study sites, as indicated by the frequency of each species; *An. sinensis*, *An. pullus*, *An. kleini*, *An. sineroides*, *An. belenrae*, and *An. lindesayi* accounted for 44.2% (118/267), 0.7% (2/267), 0.7% (2/267), 22.1% (59/267), 1.9% (5/267), and 30.3% (81/267), respectively (Table 2 and Additional file 1: Table S1). As shown in Fig. 3, *An. sinensis* numbers collected in Wologt-myeon in August decreased in September 2022. In contrast, *An. lindesayi* showed the peak in September 2022. The total number of *An. belenrae* increased in September but declined in October, while *An. sineroides* was found only in August 2022. In 2023, there was a difference in larvae population between the two breeding sites; the majority of larvae and adults was *An. lindesayi* in Wolgot-myeon during July and August. However, *An. sinensis* accounted for the highest number of adults in Naega-myeon, while *An. sineroides* accounted for the highest larvae number. *An. sinensis* larvae could not be collected in Naega-myeon.

**Table 2:** Identification of *Anopheles* spp. collected from different places in South Korea, 2022–2023

Place	Stage	PCR-positive					
		*An. sinensis*	*An. pullus*	*An. kleini*	*An. sineroides*	*An. belenrae*	*An. lindesayi*
Wolgot-myeon	Larva	1					57
	Adult	28	1	2	2	3	24
# of larva and adult		29	1	2	2	3	81
# of identification (%)		24.6 (29/118)	0.8 (1/118)	1.7 (2/118)	1.7 (2/118)	2.5 (3/118)	68.6 (81/118)
Naega-myeon	Larva				55		
	Adult	89	1		2	2	
# of larva and adult		89	1		57	2	
# of identification (%)		59.7 (89/149)	0.7 (1/149)		38.3 (57/149)	1.3 (2/149)	
# of total larva and adult		118	2	2	59	5	81
# of total identification (%)		44.2 (118/267)	0.7 (2/267)	0.7 (2/267)	22.1 (59/267)	1.9 (5/267)	30.3 (81/267)

All *Anopheles* adults in this study showed a negative result for *P. vivax* (Additional file 1: Figure S3). The probe could differentiate *P. vivax* from other species, confirming that *P. vivax* was absent in *Anopheles* adults, but infection by other *Plasmodium* species remained uncertain.

As shown in Fig. 3, *An. sinensis* numbers collected in Wologt-myeon in August decreased in September 2022. In contrast, *An. lindesayi* showed the peak in September 2022. The total number of *An. belenrae* increased in September but declined in October, while *An. sineroides* was found only in August 2022. In 2023, there was a difference in larvae population between the two breeding sites; most larvae and adults were *An. lindesayi* in Wolgot-myeon during July and August. However, *An. sinensis* accounted for the highest number of adults in Naega-myeon but *An. sineroides* accounted for the highest larvae number. *An. sinensis* larvae were not collected in Naega-myeon.

### Climate environment at breeding sites in 2022 and 2023

Four key climatic variables, relative relief, temperature, humidity, and rainfall, are known to be critical determinants of malaria incidence [44–46]. Among them, variability in malaria trends has been associated with changes in temperature and rainfall patterns, the main drivers of malaria transmission [47]. South Korea has four distinct seasons (spring, summer, autumn, and winter) and the average annual precipitation is around 1300 mm, and is especially heavy in July and August because of the rainy season [48].

In China, the temperatures between 16 and 31 °C were reported to be optimal for the complete development of *An. sinensis,* and below 16 °C, the embryonic development was hampered [49]. In South Korea, the optimal temperature for the complete development of *An. sinensis* was not reported yet, but the maximal temperature ranged up to 34.3 °C during July to October 2008 to 2012.

**Fig. 4:**
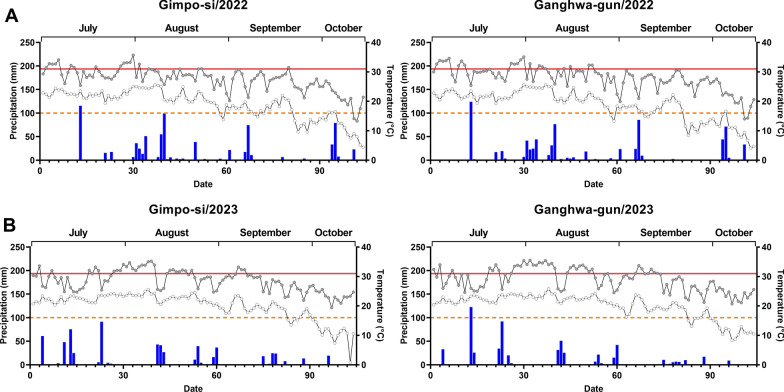
Climate change in Gimpo-si (Wolgot-myeon) and Ganghwa-gun (Naega-myeon). The vertical bars on the left side show the cumulative 104-day precipitation (blue) and temperature on the right side (maximum temperature, grey circle; minimum temperature, white circle) from July to mid-October in 2022 (**A)** and 2023 (**B**). Temperate zones of mosquito development between 16 °C (orange, dotted line) and 31 °C (red, solid line)

Therefore, the number of optimal days for mosquito development was counted using the daily maximum temperature (T_max_) and minimum temperature (T_min_) at both breeding sites to determine their suitability for larvae and adults (Fig. 4). According to the Korea Meteorological Administration, National Climate Data Center, South Korea, the two breeding sites shared a similar climate pattern of temperature range and precipitation amount.

In Gimpo-si, the total number of days with the most suitable temperature ranges (T_min:_ 16 °C ≤ and ≤ T_max_: 31 °C) were estimated to be 63 [15.8 ± 8.9 (mean ± SD)] and 59 [14.8 ± 9.0 (mean ± SD)] in 2022 and 2023, respectively (Table 3). In Ganghwa-gun, the total number of days with the most suitable temperature ranges (T_min:_ 16 °C ≤ and ≤ T_max_: 31 °C) were estimated to be 57 [14.3.8 ± 7.8 (mean ± SD)] and 47 [11.8 ± 7.2 (mean ± SD)] in 2022 and 2023, respectively. This indicates that the number of days of suitable temperature was less in 2023 than in 2022 (*P* > 0.05).

**Table 3:** Distribution of climate factors at breeding places in South Korea in 2022–2023

Place	Range of temperature	2022							2023						
		Jul	Aug	Sep	Oct^a^	Total	Mean	SD	Jul	Aug	Sep	Oct^a^	Total	Mean	SD
Gimpo-si	T_max_^b^: 31 °C <	13^a^	2	1	0	16	4	5.2	8	16	3	0	27	6.8	6.1
	T_min_^c^: < 16 °C	0	2	14	9	25	6.3	5.6	0	0	6	12	18	4.5	5.0
	T_min_: ≥ 16 °C & T_max_: ≤ 31 °C	18	27	15	3	63	15.8	8.9	23	15	21	0	59	14.8	9.0
	Total precipitation (mm)	202.5	352	118.5	147	820	205	90.1	330.5	228	97.5	23	627	169.8	118.3
	Precipitation^d^ (mm)	6.5	11.4	4.0	12.3	34.1	8.5	3.4	10.7	7.4	3.3	1.9	23.2	5.8	3.5
Ganghwa-gun	T_max_: 31 °C <	13	8	0	0	21	5.3	5.5	13	18	0	0	31	7.8	8.0
	T_min_: < 16 °C	0	2	13	11	26	6.5	5.6	0	1	13	12	26	6.5	6.0
	T_min_: ≥ 16 °C & T_max_: ≤ 31 °C	18	21	17	1	57	14.3	7.8	18	12	17	0	47	11.8	7.2
	Total Precipitation (mm)	225	298.5	128	157.5	809	202.3	65.8	344.5	205	68.5	11.5	629.5	157.4	128.9
	Mean precipitation (mm)	7.3	9.7	4.3	13.1	34.3	8.6	3.2	11.1	6.6	2.3	1.0	21.0	5.2	4.0

^a^Data were obtained until October 12th

^b^Daily maximum temperature

^c^Daily minimum temperature

^d^Mean of daily precipitation

Similarly, total precipitation (mm) in Wologot-myeon from July to October 2023 was 627 mm [5.8 ± 3.5 (mean ± SD)], which was less than 820 mm [8.5 ± 3.4 (mean ± SD] during the same period in 2022. The precipitation of Naega-myeon had a similar amount to that of Wolgot-myeon, showing 809 [8.6 ± 3.2 (mean ± SD)] and 629.5 [5.8 ± 3.5 (mean ± SD)] mm in 2022 and 2023, respectively.

Days with the most suitable temperature were highest in August (*n* = 27), 2022 in both breeding sites. However, both sites showed fewer days of optimal temperature in August than in July or September 2023 due to an increase in T_max_ of above 31 °C_._, indicating that the high temperature may adversely affect mosquito development. Overall, compared to 2022, the number of larvae in the Wolgot-myeon breeding spot decreased in 2023 (Table 1).

While the small pools of water created by rain provide habitats for eggs, larvae, and pupas to develop, excess precipitation may reduce mosquito abundance through the flushing effect of precipitation [48]. The cumulative precipitation from July to October, especially in August, was higher in 2022 than in 2023.

The meadow breeding site at Naega-myeon in Ganghwa-gun maintained a water level of about 5 cm depth in August 2023. However, although precipitation decreased from 205 to 68.5 mm between August and September, the number of larvae in the shallow freshwater in Naega-myeon increased in September (*n* = 90) compared to August (*n* = 5), indicating the involvement of additional environmental variables in larval development during September and early October. Also, the highest number of adults from cow shed was collected in August (*n* = 190), declining in September (*n* = 99), and contained the majority of *An. sinensis*, indicating that the shallow freshwater at the meadow breeding site might be less associated with the adult of *An. sinensis* captured at the cattle shed. Since the breeding spot located in the freshwater marsh of Naega-myeon was very shallow and, therefore, would be affected by precipitation, an alternative breeding site for *An. sinensis* in Naega-myeon must be continuously searched.

Especially, there was a significantly different larval distribution of *Anopheles* spp. in October 2023; *An. lindesayi* was in the majority in Wolgot-myeon and *An. sineroides* in Naega-myeon (Additional file 1: Figure S4).

### Geo-ecological environments at mosquito breeding sites

**Fig. 5:**
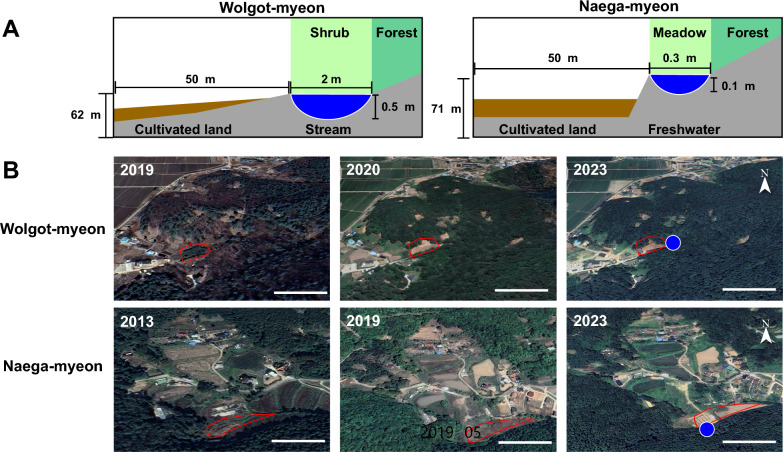
Grade map of the cultivation history of nonagricultural areas near the breeding sites during the last few years. **A** Estimated length, depth, and distance of newly cultivated land (brown color) from two breeding sites (blue color). **B** Ground lands and dwelling houses in Wolgot-myeon and Naega-myeon recorded by Google Earth Pro in different years. Scale bar = 50 m

The Wolgot-myeon breeding site was a stream at a cul-de-sac and the cultivated land was placed within 50 m distance in front of the breeding site. The stream site of 0.5 m/2 m/62 m depth/width/altitude was in a forest shrub with a little slope (less than 10°-degree angle) from ground land (Fig. 5A). The freshwater breeding site in the wet meadow of Naega-myeon was also located in a forest marsh near cultivated land with 0.1 m/0.3 m depth/width but a high elevation of up to 71 m from the ground land.

Investigation of the cultivation history of the breeding site, including the geographical change of the land use at breeding sites, might help define the next breeding site using Google Maps recording of the past images before cultivation (2019 for Wolgot-myeon and 2013 for Naega-myeon) and after cultivation (2020 for Wolgot-myeon and 2019 for Naega-myeon) (Fig. 5B). According to the past history depicted by Google Earth, the cultivated land of Wolgot-myeon was originally a mountain area until 2019 and changed to cultivated land since 2020. The agricultural land of the breeding site at Naega-myeon was originally a mountain in 2013 but has been used for cultivation since 2019.

### Distribution of breeding sites in the valley

Understanding the epidemiology of malaria transmission and variations occurring within areas with proximity to the highlands would support improving an area-specific national strategy plan for prevention and transmission control. Therefore, updating the existing geographical characteristics of breeding sites would be helpful for efficiently implementing vector control.

**Fig. 6:**
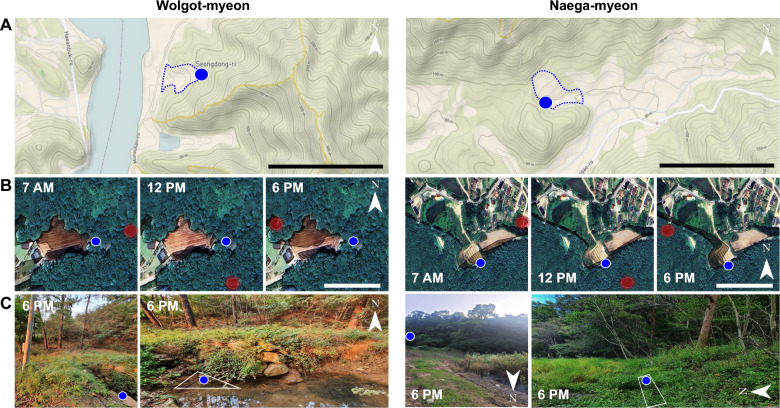
Sunlight and shadow position at larval habitats. **A** The breeding sites (blue circles) of Wolgot-myeon and Naega-myeon are surrounded by two hilly mountains, creating a valley. The black scale bar indicates 1 km. Lands depict a range of elevation in meters above mean sea level using The National Geologic Map Database (https://ngmdb.usgs.gov/ngmdb/ngmdb_home.html). The ground area of the valley (blue dot-lined zone), including breeding sites (within a 50 m contour line), was calculated by circumference line using Google Earth tools. **B** Sun (red circle) posed at that time, and thus, dark shades generated by altitude area at mountain ridge line at each time are depicted using Shadow Calculator (http://shadowcalculator.eu). The white scale bar indicates 0.1 km. **C** The shadow was confirmed at each spot by taking a picture at 6 PM

As seen in Fig. 6A, breeding sites are located inside valleys surrounded by two mountains (the mountain height above mean sea level: 150–300 m) on the north and south sides. The blue dot-lined zones are the areas (19,394 m^2^) for Wolgot-myeon and Naega-myeon (83,830 m^2^) surrounded by the valley containing the breeding sites.

Due to the interposition between valleys, the breeding sites may not be exposed to the sun directly, maintaining the shadow during the day time (Fig. 6B). Although the Wolgot-myeon breeding site was partially exposed to the sun during sunset at about 6 PM according to the sunlight and shadow information, the larvae stayed under the shade and were not directly exposed to the sun. The larvae were not detected in other surface areas of the same stream, indicating that open water surface under direct sunlight may not be suitable for *Anopheles* spp. larvae development.

According to the Shadow Calculator, the shadow area of the breeding site at Naega-myeon was not as wide as that of Wolgot-myeon. The slope of the mountain hill at Naega-myeon was 150 m above sea level compared to 300 m of the mountain at Wolgot-myeon. However, the Naega-myeon meadow maintained the shadow under the forest boundary, and the shallow freshwater was located following the edge line of the shadow where the larvae were found.

The sunlight level and shadow at the breeding site was confirmed in the afternoon at 6 PM because the breeding site at Wolgot-myeon was exposed to sunlight according to the Shadow Calculator (Fig. 5B). The edge line of the stream at Wolgot-myeon was not exposed to sunlight due to its position (Fig. 5C). Likewise, the meadow at Naega-myeon may be affected by the sunlight in the afternoon with the hottest temperature in August critically damaging larvae development in the shallow marsh. However, there might be more shade in September, as confirmed by the shadow at 6 PM (Fig. 5C), increasing larvae numbers in Naega-myeon. The time series of shade during July and October are presented in Additional file 1: Figures S5 and S6.

### Sequence-based identification of *Anopheles* species

Morphological identification of many specimens entails tedious effort [50]. DNA barcoding is an increasingly popular molecular method for identifying animal species based on partial mitochondrial DNA sequences [51]. Generally, *An. sinensis* was differentiated from its morphologically indistinguishable sibling species (e.g., *An. lesteri, Anopheles yatsushiroensis*) by comparing sequence data from the second internal transcribed spacer (ITS2) region and deciphering evolutionary relationships of the new *Anopheles* mosquitoes with the mosquitoes from neighboring countries [52].

Therefore, the phylogenetic position of *An. sinensis* from South Korea within the Hyrcanus group was determined by phylogenetic relationships using the maximum likelihood and Tamura-Nei substitution model with rate uniformity and pattern homogeneity. The phylogenetic tree was built with 1000 bootstrap replicates to investigate ITS2 and COI genetic diversity. GenBank Accession # of ITS2 and raw sequence of *Anopheles* spp. are shown in Additional file 1: Table S1.

**Fig. 7:**
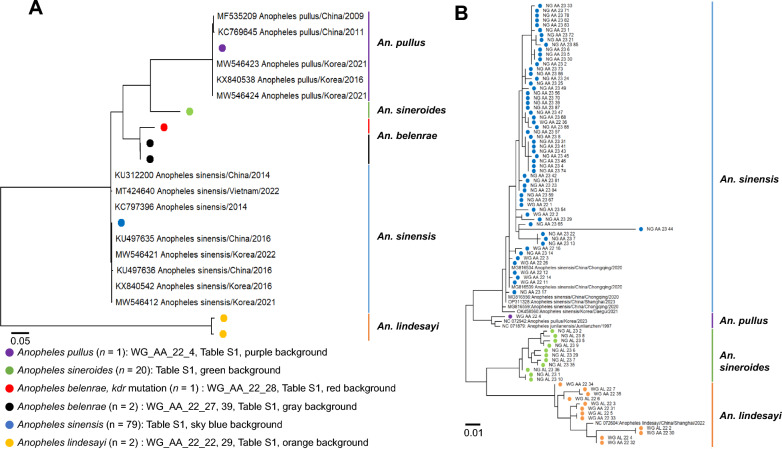
Phylogenetic tree of ITS2 and COI genes of *Anopheles* mosquitoes. **A** ITS2 genes and **B** COI genes of *Anopheles* spp*.*, constructed using the maximum likelihood method with 1000 bootstrap iterations using the MEGAX tool. (https://www.megasoftware.net/). Cut off bootstrap values area was > 70. *WG* Wolgot-myeon, *NG* Naega-myeon, *AA* *Anopheles* adults, *AL* *Anopheles* larvae

As shown in Fig. 7A, the ITS2 barcode sequences of *Anopheles* spp. [group of *An. sinensis* (*n* = 79) and *An. pullus* (*n* = 1)] were clustered in one lineage close to the old strains of each *Anopheles* species found in South Korea and China*.* Interestingly, two of three *An. belenrae* isolates maintained an extremely conserved sequence [451/451(100%)]*.* The third *An. belenrae* isolate (GenBank Accession # of ITS2:OQ303977) was less conserved [430/451(95.34%)] than the other two isolates, indicating a potential difference of genotypes among *An. belenrae* strains circulating in Korea. Furthermore, this *An. belenrae* showed the *kdr* mutation allele for the first time in South Korea.

COI sequences of all *An. sinensis* isolates showed a high homology of more than 99%. *An. sinensis* and *An. pullus* were clustered with Korean and Chinese isolates from 2017 to 2023 within 99% genetic homology. Interestingly, *An. lindesayi*, major species of the transparent stream of Wolgot-myeon, maintained 98% sequence homology of COI, but the *An. sineroides*, major species of the turbid fresh shallow water of Naega-myeon, showed a less conserved, about 90% sequence identity than any other *Anopheles* spp. (Fig. 7B).

### Polymorphisms and geographical distribution of the knockdown resistance (*kdr*) gene of *Anopheles* spp. in South Korea

**Fig. 8:**
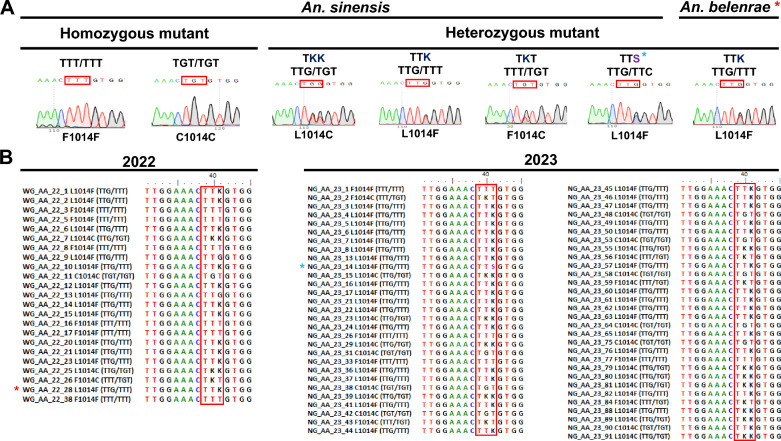
*kdr* gene polymorphism of *Anopheles* spp. at codon 1014 in South Korea. **A** Chromatogram of the DNA sequence of the *kdr* allelic mutation site in *An. sinensis* collected in this study. Red boxes indicate *kdr* mutation sites. Two homozygous mutants [TTT(F)/TTT(F) and TGT(C)/TGT(C)] and three heterozygous mutants [TTG(L)/TGT(C), TTG(L)/TTT(F), and TTT(F)/TGT(C)] are shown. K = G/T [53]; S = G/C [54]. **B** Alignments of *kdr* genotypes detected in Wolgot (WG)-myeon in 2022 and Naega (NG)-myeon in 2023. The red asterisk indicates the first report of the L/F1014 *kdr* mutation in wild-type populations of *An. belenrae.* The blue asterisk indicates an alternative L/F mutant type with TTC instead of TTT in *An. sinensis*

In South Korea, the intensive use of insecticides has contributed to the rapid development and spread of insecticide resistance in the *An. sinensis* complex, inducing six *kdr* genotypes [23, 24]. Six genotypes of *An. sinensis* were detected in Wolgot-myeon in 2022 (Table 4), including the wild-type homozygote TTG(L)/TTG(L) (8.3%); mutant homozygotes TTT(F)/TTT(F) (25.0%) and TGT(C)/TGT(C) (4.2%); and wild-type/mutant heterozygotes TTG(L)/TTT(F) (50.%), TTG(L)/TGT(C) (8.3%), and TTT(F)/TGT(C) (4.2%). *Anopheles sineroides* maintained wild-type *kdr* genotype, but for the first time, a *kdr* mutant (L/F1014) at codon 1014 was detected in *An. belenrae*. Chromatograms of DNA segments of each genotype and alignment are shown in Fig. 8A and B, respectively. Information on the *kdr* genotype of each *Anopheles* species is presented in Additional file 1: Tables S1 and S2.

**Table 4:** Screening of *kdr* mutations in the voltage-gated sodium channel (VGSC) gene in *Anopheles* species

Study sites	Year	Species	Wild type (%)		Mutant type (homozygote) (%)		Mutant type (Heterozygote) (%)			
			TTG(L)/TTG(L)	TTA(L)/TTA(L)	TTT(F)/TTT(F)	TGT(C)/TGT (C)	TTG(L)/TTT(F)	TTG(L)/TGT(C)	TTT(F)/TGT(C)	TTG(L)/TTC^b^(F)
Wolgot-myeon	2022	*An. sinensis* (*n* = 24)	8.3 (2/24)		25.0 (6/24)	4.2 (1/24)	50.0 (12/24)	8.3 (2/24)	4.2 (1/24)	
		*An. pullus* (*n* = 1)	100.0 (1/1)							
		*An. sineroides* (*n* = 2)		100.0 (2/2)						
		*An. belenrae* (*n* = 3)	66.7 (2/2)				33.3 (1 ^a^/2)			
Naega-myeon	2023	*An. sinensis* (*n* = 61)	3.3 (2/61)		6.6 (4/61)	14.8 (9/61)	49.2 (30/61)	16.4 (10/61)	8.2 (5/61)	1.6 (1^b^/61)
		*An. sineroides* (*n* = 3)		100.0 (3/3)						

^a^First report of the L/F1014 *kdr* mutation in *An. belenrae* in Wolgot-myeon

^b^TTG(L)/TTC(F) in L/F1014 *kdr*

In 2023, the majority of Naega-myeon adults was identified as *An. sinensis* (Table 1) and they showed the wild-type homozygote TTG(L)/TTG(L) (3.3%); mutant homozygotes TTT(F)/TTT(F) (6.6%) and TGT(C)/TGT(C) (14.8%); and wild-type/mutant heterozygotes TTG(L)/TTT(F) (49.2%), TTG(L)/TGT(C) (16.4%),TTT(F)/TGT(C) (8.2%), and TTG(L)/TTC(F) (1.6%). Among these six *An. sinensis* genotypes, L/F with TTT is common, but a rare L/F allele genotype was detected with TTC in this study. This rare genotype was detected in the southeast but not in the west coast area in South Korea [24].

A previous study employed the pyrethrin insecticide etofenprox in wild *Anopheles* adults in South Korea, and a lethal dose (LD_50_) of etofenprox was reported as 2.41 μg/female adult using a direct-contact mortality bioassay of 24-h exposure to etofenprox [36]. The results with the female adults derived from the captured *Anopheles* species (*An. lindesayi*) larvae at Wolgot-myeon were comparable to the previously reported results, indicating similar toxicity of etofenprox to *Anopheles* adults in the field (LD_50_ = 2.43 μg/female adult). However, adults (F1) derived from *An. sinensis* larvae by rearing in the laboratory after hatching eggs from blood-fed adults, may have two-fold better endurance to etofenprox (LD_50_ = 5.78 ± 2.28 μg/female) (mean ± SD, triplicate) (Additional file 1: Figure S7).

Furthermore, based on a previous study reporting LD_50_ of etofenprox as 1.38 mg/L on *An. sinensis* larvae [37], its effect on different *Anopheles* spp. larvae was tested. LD_50_ of *Anopheles* larvae collected from Wolgot-myeon, the majority of which in the first week of October 2023 was *An. lindesayi,* was 5.83 mg/L (*n* = 10/group). In Naega-myeon, most larvae in the first week of October 2023 consisted of *An. sineroides*, showing 6.61 ± 4.37 mg/L (mean ± SD, triplicate) as LD_50_ of etofenprox.

The third-four instars of *An. sinensis* reared from the hatched eggs in the laboratory by blood-fed females showed 17.71 mg/L (*n* = 10/group) as LD_50_ of etofenprox. As a reference, the toxicity of etofenprox to third instars of *Culex* spp. larvae from Naega-myeon, based on the 24-h exposure contact mortality bioassays, has been reported as 0.08–2.46 mg/L of LD_50_ depending on the different areas in South Korea [55], and this study of Naega-myeon larvae showed 0.22 mg/L of LD_50_ (*n* = 10/group) to *Culex territanes* larvae (Additional file 1: Figure S8).

## Discussion

Human malaria is a complex disease in which the interactions of the *Anopheles* mosquito vector with the parasite, humans, and environmental factors are involved in malaria transmission [40]. Although the pan-governmental malaria control efforts dramatically reduced *P. vivax*-derived malaria burden in South Korea over the last 10 years, it still remains endemic in 2023, with over 700 cases, including imported cases [56]. In South Korea, vivax malaria is categorized into two types based on the incubation period. Gimpo-si and Ganghwa-gun are considered important for intensive vector control because they have cases with a short incubation period of 12–17 days and a longer incubation period of 6 months involving drug-resistant patients, as reported in 2020 [56, 57].

In South Korea, *P. vivax*-infected mosquitoes were reported in DMZ in 2020 [1], but there is little information on *Anopheles* larval breeding sites near DMZ over a decade. A better understanding of geographical and seasonal distributions and anthropophilic behaviour of the vector is essential for developing an effective malaria control programme. Given the current malaria situation in South Korea, it is necessary to update information on *Anopheles* breeding sites continuously. For efficient malaria control, understanding the environmental conditions of the habitats of *Anopheles* larvae and adults would help improve vector clearance by eradicating the larvae breeding sources [58]. Information on the spatial and temporal distribution of *Anopheles* larvae is a prerequisite for devising practical and sustainable malaria vector control methods that target immature mosquito stages [59]. However, larval surveys are frequently costly, time-consuming, and cumbersome.

An alternative remote sensing in a geographical information system was reported in 2005 that could evaluate land use correlated with *Anopheles* habitats, monitoring rice fields in Paju and Ganghwa-gun areas in South Korea [40]. Mosquito larvae were found in rice fields, open grassy ponds, ground pools, swamps, marshes, lake shores, stream margins, and ditches [17]. These are generally fresh, shallow water habitats in China and South Korea, either stagnant or flowing, usually with emergent vegetation, and exposed to sunlight [15, 17]. Though larvae numbers collected in rice fields were smaller than those collected in parsley fields and marshes, the main breeding place of *An. sinensis* was undoubtedly the rice field. However, no additional breeding sites in South Korea have been reported since 2007 compared with China, which received a malaria-free certification from the World Health Organization (WHO), a notable feat for a country that reported 30 million cases annually in the 1940s [60]. Also, malaria cases in Japan have decreased rapidly to below 20 cases annually since the Zero Malaria 2030 campaign was launched in 2017 to end malaria [61, 62]. Although the largest area of agricultural land is still the rice paddy in South Korea, there may be changes heavily impacting the distribution and density of larval populations over the mosquito season.

In 2007, two US army groups reported the last information in the joint security DMZ areas and several different provinces on breeding sites in South Korea [16, 18]. Based on these reports, *An. sinensis* accounted for the highest proportion of all larvae (36.4%–58.6%) in all habitats, and stream margins were relatively less preferable breeding spots. However, *An. kleini* accounted for the highest proportion at the stream margins in South Korea in July [18]. Stream locations and ground pools were also positive for *An. lindesayj* larvae in Gyeongsangnam, Chungcheongbuk, and Gyeonggi Provinces [63]. The high abundance of *An. lindesayi* larvae at the stream margin of Wolgot-myeon in this study was in agreement with the previous report [18]. Habitats of *An. sineroides* larvae were reported as depression, ditch, and marsh but not stream margins, indicating that the observation at Naega-myeon was consistent with the previous report [18].

There was a difference in the number of *Anopheles* spp. larvae and adults in two breeding sites. *An. kleini* accounted for the highest proportion of *Anopheles* spp. in June and July, and *An. pullus* in May and June [1, 16]. In this study, larvae and adults were collected during August and September, likely leading to low numbers of *An. kleini* and *An. pullus*. In contrast, the mean larval population of *An. sinensis* was reported to be low in May, but the mean numbers increased through August at the end of the wet monsoons, declining in September as habitats dried up due to rice harvesting in the DMZ area in 2007 and 2020 [1, 18]. Thus, the study performed in Wolgot- myeon and Naega-myeon was consistent with these reports.

Historically, *An. sinensis* and *An. sineroides* larvae in Ganghwa-gun were abundant in rice paddies and flooded areas, respectively, as reported in 2005 [40], implicating that the *An. sineroides* breeding site might have been a breeding habitat in Naega-myeon for a long time. Besides, consistent with the observation in Wolgot-myeon, *An. lindesayi* was found in steam margins in South Korea [64]. However, the relatively high abundance of *Anopheles* spp. near the DMZ area and low abundance in southern Seoul were difficult to explain because of the similarity of habitats and farming practices throughout South Korea, implicating the involvement of additional ecological variables in the breeding sites. These parameters include chemical (pH, chemical oxygen, ammonia, nitrogen, sulfate, and chloride) and biological (floating plants, predators, algae cover, and aquatic vegetation) factors [15], which must be further studied.

The breeding sites in this study, Wolgot-myeon and Naega-myeon, were distinct from each other. Although cattle sheds have long been associated with a high abundance of *An. sinensis* vector density in South Korea and China [17, 29, 65], they were not found in a 2 km radius in Wolgot-myeon, while the breeding site of Naega-myeon was close to cattle sheds within 100 m distance. As *An. sinensis* had limited dispersal, with most adults staying close to their larval habitats, and dispersal distances were less than 1 km [49], human blood would be the main resource for oviposition at Wolgot-myeon but not Naega-myeon.

Previously, the monthly average temperature and precipitation amount have been shown to correlate significantly with the average abundance of mosquitoes in South Korea [66]. Mosquitoes, like other ectotherms, are highly susceptible to changes in ambient temperature, demonstrably affecting their growth, reproduction, metabolic rate, lifespan, biting rate, and immunity [67]. The mosquito abundance could be determined by successful embryogenesis and hatching of eggs. Depending on the mosquito species and stage of development, the lower temperature limits were between 5–10 °C while the upper temperature limits were between 30°–35 °C [8].

In China, *An. sinensis* development could occur at temperatures as low as 16 °C but not at temperatures > 31 °C [49]. Especially at 25–30 °C, *An. sinensis* hatched within 3 days after oviposition but below 16 °C, embryonic development of the *An. sinensis* could not be completed in China [49]. Based on the analysis of temperature and precipitation during the period from 23 to 43 weeks in 2008–2012 in South Korea, the means of T_max_/T_min_/T_mean_ temperatures and precipitation were 26.9 ± 3.8/16.6 ± 5.59/21 ± 4.63 °C/8.7 ± 13.57 mm (mean ± SD) for *An. sinensis* abundance [48]. It implies that *Anopheles* would not spread through South Korea, but potentially their habitats could move to the more suitable temperature/humidity areas according to climate change followed by global warming.

Increasing the temperature by 1 °C in the range of 18–26 °C increases the life span of a mosquito by more than a week [68]. In this study, the number of larvae in Wolgot-myeon was reduced in 2023 compared to 2022, suggesting that the change in T_min_ was less relevant than T_max_ for abundance of larvae because the total number of days below 16 °C in 2023 was decreased from 25 to 18 days in Wolgot-myeon, implying that the larvae may endure low temperatures (16 °C <) better than at high temperatures (> 31 °C).

In this study, the total rainfall of Wolgot-myeon was lower in 2023 than in 2022 during July to the middle of October, which was unfavorable for larvae development. Previously, the daily precipitation (mm) for mosquito abundance was 8.7 ± 13.57 mm (mean ± SD) in South Korea during July to September during 2008 and 2012 [48]. The mean weekly precipitation (mm) from July to October 2022 was comparable to the previous report, showing 8.5 ± 3.4 (mean ± SD) mm, while it was 5.8 ± 3.6 mm (mean ± SD) in 2023. The mean of daily precipitation of Naega-myeon showed 5.2 ± 4.0 (mean ± SD) mm in 2023, indicating that Naega-myeon would have less humidity than Wolgot-myeon. Therefore, hotter temperatures and lower precipitation levels in 2023 might be the reasons for reduced *Anopheles* larvae abundance in Wolgot-myeon.

Accurately predicting malaria vector occurrence and *Plasmodium* transmission risk in heterogeneous environments is essential to permit region-specific, cost-effective intervention strategies and heightened surveillance [69]. Environmental factors, including land cover [70] and land use, such as farming, deforestation [71], and altitude of house > 500 m above sea level [72], have been previously associated with malaria incidence in endemic areas and are, therefore, relevant in designing an effective targeted malaria control programme.

Topography (e.g., hills, valleys, elevation) of terrain has long been recognized to be one of the factors associated with malaria [73] due to its association with cooler temperatures that affect the development of *Anopheline* vectors and the *Plasmodium* parasites [74]; 25 °C was predicted as optimal malaria transmission. However, the impact of the terrain topography, cultivation, agriculture, mosquito diversity, and malaria transmission in South Korea is poorly documented. The current breeding sites of *Anopheles* larvae in Wolgot-myeon and Naega-myeon were near the mountain area, implicating the potential upward movement of breeding sites into the shade to avoid hot temperatures enhanced by cultivation. Although Naega-myeon may be considered a transient breeding spot due to the shallow freshwater, the topography of the terrain located in the valley has the potential of a huge breeding site for mosquito breeding in September to October than in August based on the suitable temperature and humidity under the deep shade.

Light intensity is one of the multiple variables associated with larvae of *Anopheles* spp. and shade was reported to be a positive environmental factor for *Anopheles gambiae* [59]. Consistent with this observation, two current breeding sites in this study were not exposed to sunlight. In Wolgot-myeon, all larvae stayed at stagnant places under the shade of a stream margin, inconsistent with a previous report that *Anopheles* larvae were found in areas exposed to sunlight in South Korea [17].

Furthermore, there was a distinct difference in larvae species between the end of September and early October. Compared to 2022, there was a significantly different distribution of *Anopheles* spp. in October 2023, with the majority of *An. lindesayi* in the transparent stream water of Wolgot-myeon and *An. sineroides* in the turbid and shallow freshwater in Naega-myeon (Additional file 1: Figure S4). Interestingly, larvae were not found at Wolgot-myeon stream after early October in 2022, but 1st and 2nd instars were most frequently collected in the static shaded stream of Wolgot-myeon in 2023 (mostly *An. lindesayi*) from early to middle of October. The absence of larvae at Wolgot-myeon in 2022 could be due to the damage to egg hatching rather than impaired larvae growth by a sudden drop in temperature to below 16 °C for 14 days between the middle and end of September 2022 (Fig. 4). In 2023, T_min_ dropped below 16 °C for only 6 days in the end of September. In contrast, slow-growing L3-L4 larvae were observed in Naega-myeon until the first week of October with a T_min_ below 16 °C. L1–L2 instars larvae were not detected during this period, indicating that, compared to egg hatching, larvae growth might be less impaired at lower temperatures.

In addition to the effect of climate change on the population and the number of mosquitoes, the increased resistance against pesticides could affect the change in the number of mosquitoes in the field. In South Korea, pyrethroids such as etofenprox, deltamethrin, lambda-cyhalothrin, permethrin, and cyfluthrin have been widely used as insecticide and pesticide groups [23, 75]. The knockdown resistance (*kdr*) has been frequently reported in pyrethroid-resistant *An. sinensis* in Gimpo-si and was prevalent with F/F mutant type in 2016, but in this study, the most common *kdr* allele frequency of *An. sinensis* was the L/F mutant type, implicating active dynamic change of the *kdr* mutation frequency in South Korea [23, 24]. Besides, for the first time, a rare TTG (L)/TTC (F) allele genotype in *An. sinensis* was detected in Ganghwa-gun, previously reported in southeast of South Korea in 2012 [24]. The TTG (L)/TTC (F) was also found in Anhui, China, 2019 [54], supporting an expansive development and spread of insecticide resistance polymorphism in *An. sinensis.* The *kdr* mutant allele was found only in *An. sinensis* but not in the other four species (*An. pullus, An. kleini, An. lesteri,* and *An. belenrae*) [23, 24]. In this study, for the first time, the first *kdr* mutation in *An. belenrae* at codon 1014 (L/F1014) was detected, which was the most frequent mutant allele in *An. sinensis* (Additional file 1: Table S2).

The resistance of *Anopheles* spp*.* to pyrethroid insecticide was examined by measuring LD_50_ of etofenprox in *Anopheles* captured at two breeding sites. LD_50_s of the larvae captured at Wolgot-myeon and Naega-myeon were 5.83 mg/L and 6.61 mg/L, indicating that at least 4 folds higher than the previously reported LD_50_ (1.38 mg/L) [37] (Additional file 1: Figure S7 and S8). Interestingly, third-fourth instars of *An. sinensis* reared from the hatched eggs in the laboratory by blood-fed females showed 17.71 mg/L (*n* = 10/group), a tenfold higher etofenprox LD_50_ than previously reported [37].

There were several limitations of this study. *Anopheles* larvae derived from the oviposition of blood-fed females at cow sheds or the field-collected larvae were used for conducting the etofenprox bioassay. Following WHO guidelines [35], non-blood-fed 2–5-day-old female mosquitoes had to be used, but collecting them in the field was difficult. Besides, rearing *An. sinensis* to develop into adults in the laboratory was not efficient, causing the low number of sample size in the bioassay. Among the limitations of this study, a small number of mosquito adults were analysed at each breeding site. Only 5 traps were installed at Wolgot-myeon for collection of adults, resulting in much fewer adults in this field study than in the previous study during 2008–2012 [48]. In Naega-myeon, the cow shed showed 40 blood-fed adults per trap in the second week of September 2023. The low abundance of adults in Wologot-myeon might be because it was far from the cattle shed but maintained a few adults between a stream near the forest and human residential areas. The traps were installed in some households 100 m from the Wolgot-myeon breeding site and those houses had already routinely installed UV night traps during the mosquito season (Additional file 1: Figure S9), causing the low number of captured adults relative to larvae.

Another drawback of the study was that larva species identification was not conducted at the time of collection because larvae were positive for *Anopheles* spp. by morphology (Additional file 1: Figure S4). Flat positioning of the larvae parallel to the water surface and the absence of siphon were used to identify *Anopheles* larvae as previously reported [59], and species identification was performed after the larvae developed into adults. Furthermore, genomic DNA was extracted from frozen larvae after one year, resulting in suboptimal DNA recovery and inefficient identification of all larvae spp.

Also, the probe used could only detect *P. vivax* in mosquitoes but no other species. These results indicated that *P. vivax* was absent, providing no information on other *Plasmodium* species in the infected adults. In the future, other breeding sites inside the same valley area should also be examined with a focus on multiple variables, such as wind speed, humidity, grass temperature, and the lowest temperature reached overnight [48].

## Conclusion

In summary, two larval breeding sites of *Anopheles* spp. were identified at the stream margin and the shallow freshwater in the meadow located near a forest in South Korea. For the first time, the occurrence of *kdr* mutation in *An. belenrae* was reported and significantly, *kdr* mutation dynamics in *An. sinensis* were reported in South Korea.

### Supplementary Information

**Additional file 1: Figure S1.** Study sites near DMZ in 2022. **Figure S2.** *Anopheles* spp. larva in Wolgot-myeon breeding site in 2022. **Table S1.** Raw sequence of Anopheles mosquitoes. **Table S2.** Raw *kdr* gene sequence with mutant genotype (L1014F) of *An. belenrae* (GenBank accession #: OQ303977). **Figure S3.** RT-PCR result of *Anopheles* adults for detection of *Plasmodium vivax*. **Figure S4.** Morphology of the *Anopheles* spp. larvae in two breeding sites in 2023. **Figure S5.** Time-series of shade of sun light from June to October in breeding site of Wolgot-myeon. **Figure S6.** Time-series of shade of sun light from June to October in breeding site of Naega-myeon. **Figure S7.** Toxicities of insecticide (Etofenprox of Pyrethroid group) to adults of Anopheles spp. **Figure S8.** Toxicities of insecticide (Etofenprox of Pyrethroid group) to larvae of Anopheles spp. **Figure S9.** Electronic mosquitoes trap (arrow) installed by host in human dwelling in Wolgot-myeon.

## Data Availability

All materials described in the manuscript, including all datasets, can be accessed by any scientist wishing to use them for non-commercial purposes. Novel DNA sequences of ITS2, COI, and *kdr* were deposited in NCBI, a part of repositories of International Nucleotide Sequences Collaboration, and GenBank accession numbers appear on additional information.
