# Supplementary material for: Identification of breeding habitats and kdr mutations in Anopheles spp. in South Korea
Source: Malar J. 2023 Dec 16;22:381. doi: 10.1186/s12936-023-04821-x (PMC10724954; doi:10.1186/s12936-023-04821-x)
Supplement: Supplementary file 1 — Additional file 1: Figure S1. Study sites near DMZ in 2022. Figure S2. Anopheles spp. larva in Wolgot-myeon breeding site in 2022. Table S1. Raw sequence of Anopheles mosquitoes. Table S2. Raw kdr gene sequence with mutant genotype (L1014F) of An. belenrae (GenBank accession #: OQ303977). Figure S3. RT-PCR result of Anopheles adults for detection of Plasmodium vivax. Figure S4. Morphology of the Anopheles spp. larvae in two breeding sites in 2023. Figure S5. Time-series of shade of sun light from June to October in breeding site of Wolgot-myeon. Figure S6. Time-series of shade of sun light from June to October in breeding site of Naega-myeon. Figure S7. Toxicities of insecticide (Etofenprox of Pyrethroid group) to adults of Anopheles spp. Figure S8. Toxicities of insecticide (Etofenprox of Pyrethroid group) to larvae of Anopheles spp. Figure S9. Electronic mosquitoes trap (arrow) installed by host in human dwelling in Wolgot-myeon. [file 12936_2023_4821_MOESM1_ESM.docx]

**Identification of breeding habitats and *kdr* mutations in *Anopheles* spp. in South Korea.**

**Supplementary Figures and Table**

**Fig. S1. Study sites near DMZ in 2022**

**Fig. S2. *Anopheles* spp. larva in Wolgot-myeon breeding site in 2022**

**Table. S1. Raw sequence of Anopheles mosquitoes**

**Table S2. Raw *kdr* gene sequence with mutant genotype (L1014F) of *An. belenrae* (GenBank accession #:** OQ303977)

**Fig. S3. RT-PCR result of *Anopheles* adults for detection of *Plasmodium vivax***

**Fig. S4. Morphology of the *Anopheles* spp. larvae in two breeding sites in 2023.**

**Fig. S5. Time-series of shade of sun light from June to October in breeding site of Wolgot-myeon.**

**Fig. S6. Time-series of shade of sun light from June to October in breeding site of Naega-myeon.**

**Fig. S7. Toxicities of insecticide (Etofenprox of Pyrethroid group) to adults of Anopheles spp..**

**Fig. S8. Toxicities of insecticide (Etofenprox of Pyrethroid group) to larvae of Anopheles spp..**.

**Fig. S9. Electronic mosquitoes trap (arrow) installed by host in human dwelling in Wolgot-myeon.**


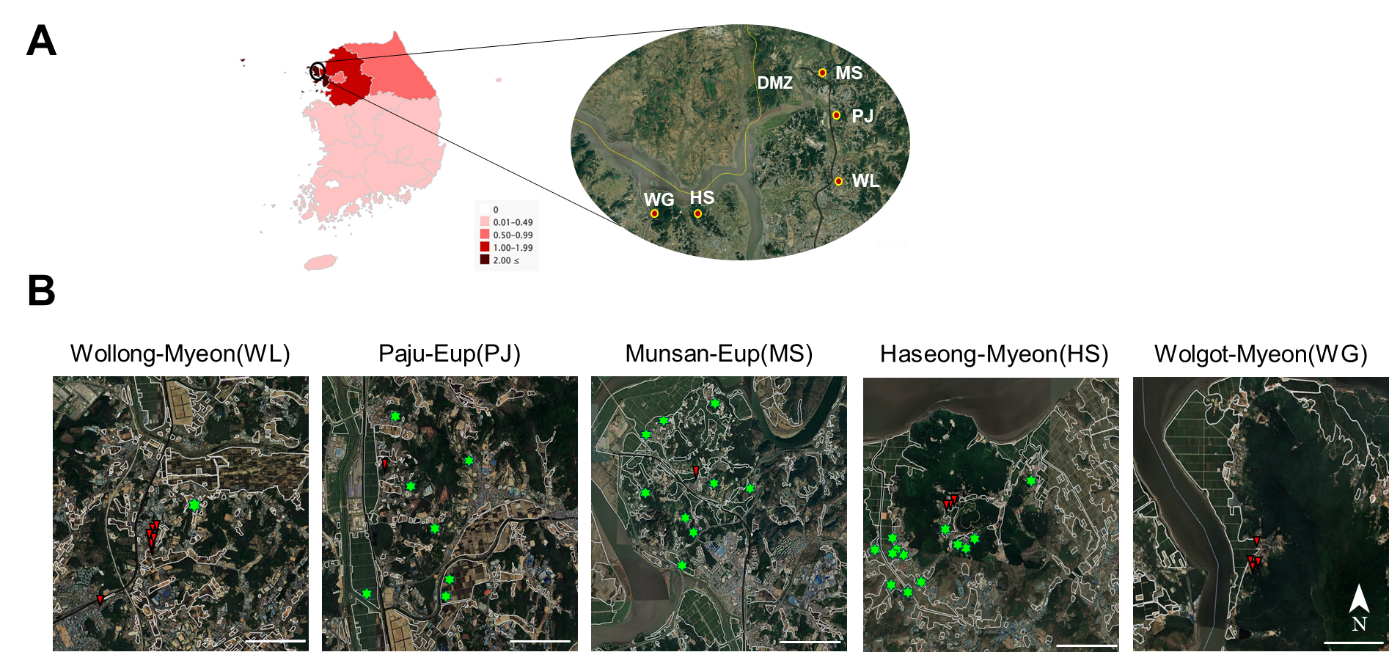


**Fig. S1. Study sites near DMZ in 2022.** Study was conducted between May and October 2022 at Wollong-myeon (37°777 - 37°782’ N, 126°791’- 126°792’ E), Paju-eup (37°837’ N, 126°795’ E), and Munsan-eup (37°883’ N, 126°774’ E), Goyang City, Gyeonggi-do and Haseong-myeon (37°742 - 37°743’ N, 126°595’- 126°596’ E) and Wolgot-myeon(37°745’ N, 126°532’- 126°535’ E), Gimpo City, Gyeonggi-do. Scale bar = 1 km. (Green star, cattle shed; red arrowhead, collection site of larvae and adult of mosquitoes; white line, rice field). Except HS and WG, we could not find the breeding sites.

**
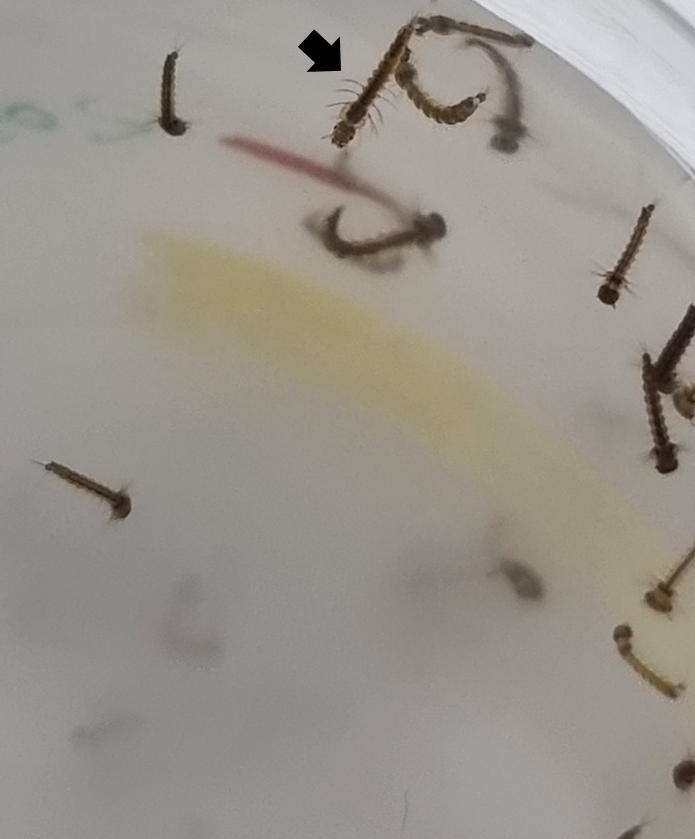
**

**Fig. S2. *Anopheles* spp. larva in Wolgot-myeon breeding site in 2022**

**Table. S1. Raw sequence output of *Anopheles* mosquitoes.** Background color indicates the group of each *Anopheles* spp. used for phylogenetic tree of ITS2 of Fig. 7.

1. **Larvae at Wolgot-myeon in 2022**

| ***#*** | ***Anopheles* spp.** | **Date** | **Place** | **Method of Identification** |
| --- | --- | --- | --- | --- |
|  |  |  |  |  |
| **WG_AL_22_1** | ***An. sinensis*** | **2022.10.06-07** | **WG** | **ITS2** |
| **WG_AL_22_2** | ***An. lindesayi*** | **2022.10.06-07** | **WG** | **COI** |
| **WG_AL_22_3** | ***An. lindesayi*** | **2022.10.06-07** | **WG** | **COI** |
| **WG_AL_22_4** | ***An. lindesayi*** | **2022.10.06-07** | **WG** | **COI** |
| **WG_AL_22_5** | ***An. lindesayi*** | **2022.10.06-07** | **WG** | **COI** |
| **WG_AL_22_6** | ***An. lindesayi*** | **2022.10.06-07** | **WG** | **COI** |
| **WG_AL_22_7** | ***An. lindesayi*** | **2022.10.06-07** | **WG** | **COI** |

1. **Adult at Wolgot-myeon in 2022**

| ***#*** | ***Anopheles* spp.** | **Date** | **Place** | **ITS2** | ***kdr* type** | **susceptibility (S) ^b^ or resistance (R) ^c^** |
| --- | --- | --- | --- | --- | --- | --- |
|  |  |  |  | **Accession number** |  |  |
| **WG_AA_22_1** | *An. sinensis* | **2022.08.04-05** | **WG** | **OP820327** | **TTT(F)/TTT(F)** | **R** |
| **WG_AA_22_2** | *An. sinensis* | **2022.08.04-05** | **WG** | **OP820334** | **TTG(L)/TTT(F)** | **R** |
| **WG_AA_22_3** | *An. sinensis* | **2022.08.04-05** | **WG** | **OP820335** | **TTG(L)/TTT(F)** | **R** |
| **WG_AA_22_4** | *An. pullus* | **2022.08.04-05** | **WG** | **OP902524** | **TTG(L)/TTG(L)** | **S** |
| **WG_AA_22_5** | *An. sinensis* | **2022.08.10-11** | **WG** | **OP820328** | **TTT(F)/TTT(F)** | **R** |
| **WG_AA_22_6** | *An. sinensis* | **2022.08.10-11** | **WG** | **OP820329** | **TTG(L)/TTT(F)** | **R** |
| **WG_AA_22_7** | *An. sinensis* | **2022.08.10-11** | **WG** | **OP820341** | **TTG(L)/TGT(C)** | **R** |
| **WG_AA_22_8** | *An. sinensis* | **2022.08.10-11** | **WG** | **OP820330** | **TTT(F)/TTT(F)** | **R** |
| **WG_AA_22_9** | *An. sinensis* | **2022.08.10-11** | **WG** | **OP820342** | **TTG(L)/TTT(F)** | **R** |
| **WG_AA_22_10** | *An. sinensis* | **2022.08.10-11** | **WG** | **OP820331** | **TTG(L)/TTT(F)** | **R** |
| **WG_AA_22_11** | *An. sinensis* | **2022.08.10-11** | **WG** | **OP820332** | **TGT(C)/TGT (C)** | **R** |
| **WG_AA_22_12** | *An. sinensis* | **2022.08.10-11** | **WG** | **OP820333** | **TTG(L)/TTT(F)** | **R** |
| **WG_AA_22_13** | *An. sinensis* | **2022.08.10-11** | **WG** | **OP820343** | **TTG(L)/TTT(F)** | **R** |
| **WG_AA_22_14** | *An. sinensis* | **2022.08.10-11** | **WG** | **OP820336** | **TTG(L)/TTT(F)** | **R** |
| **WG_AA_22_15** | *An. sinensis* | **2022.08.10-11** | **WG** | **OP820337** | **TTG(L)/TTT(F)** | **R** |
| **WG_AA_22_16** | *An. sinensis* | **2022.08.10-11** | **WG** | **OQ303891** | **TTT(F)/TTT(F)** | **R** |
| **WG_AA_22_17** | *An. sinensis* | **2022.08.10-11** | **WG** | **OQ303892** | **TTT(F)/TTT(F)** | **R** |
| **WG_AA_22_18** | *An. sineroides* | **2022.08.10-11** | **WG** | **OP902521** | **TTA(L)/TTA(L)** | **S** |
| **WG_AA_22_19** | *An. sineroides* | **2022.08.10-11** | **WG** | **OP902519** | **TTA(L)/TTA(L)** | **S** |
| **WG_AA_22_20** | *An. sinensis* | **2022.08.24 - 25** | **WG** | **OP820339** | **TTG(L)/TTT(F)** | **R** |
| **WG_AA_22_21** | *An. sinensis* | **2022.08.24 - 25** | **WG** | **OP820340** | **TTG(L)/TTT(F)** | **R** |
| **WG_AA_22_22** | *An. lindesayi* | **2022.08.24 - 25** | **WG** | **OQ303991** | **ND^d^** | **ND** |
| **WG_AA_22_23** | *An. sinensis* | **2022.08.31 - 09.01** | **WG** | **OP820338** | **TTG(L)/TTT(F)** | **R** |
| **WG_AA_22_24** | *An. sinensis* | **2022.08.31 - 09.01** | **WG** | **OQ303990** | **ND** | **ND** |
| **WG_AA_22_25** | *An. sinensis* | **2022.09.20-21** | **WG** | **OP820344** | **TTG(L)/TGT(C)** | **R** |
| **WG_AA_22_26** | *An. sinensis* | **2022.09.20-21** | **WG** | **OP820345** | **TTT(F)/TGT(C)** | **R** |
| **WG_AA_22_27** | *An. belenrae* | **2022.09.20-21** | **WG** | **OP902522** | **TTG(L)/TTG(L)** | **S** |
| **WG_AA_22_28** | *An. belenrae* ^a^ | **2022.09.20-21** | **WG** | **OQ303977** | **TTG(L)/TTT(F)** | **R** |
| **WG_AA_22_29** | *An. lindesayi* | **2022.09.20-21** | **WG** | **OQ303978** | **ND** | **ND** |
| **WG_AA_22_30** | *An. lindesayi* | **2022.09.20-21** | **WG** |  | **ND** | **ND** |
| **WG_AA_22_31** | *An. lindesayi* | **2022.09.20-21** | **WG** |  | **ND** | **ND** |
| **WG_AA_22_32** | *An. lindesayi* | **2022.09.20-21** | **WG** |  | **ND** | **ND** |
| **WG_AA_22_33** | *An. lindesayi* | **2022.09.20-21** | **WG** |  | **ND** | **ND** |
| **WG_AA_22_34** | *An. lindesayi* | **2022.09.20-21** | **WG** |  | **ND** | **ND** |
| **WG_AA_22_35** | *An. lindesayi* | **2022.09.20-21** | **WG** |  | **ND** | **ND** |
| **WG_AA_22_36** | *An. sinensis* | **2022.09.28-29** | **WG** | **OP820346** | **TTG(L)/TTG(L)** | **S** |
| **WG_AA_22_37** | *An. sinensis* | **2022.09.28-29** | **WG** | **OP820347** | **TTG(L)/TTG(L)** | **S** |
| **WG_AA_22_38** | *An. sinensis* | **2022.10.06-07** | **WG** | **OP820348** | **TTT(F)/TTT(F)** | **R** |
| **WG_AA_22_39** | *An. belenrae* | **2022.10.06-07** | **WG** | **OP902520** | **TTG(L)/TTG(L)** | **S** |

^a^: the first isolate of *An. belenrae* with insecticide resistance

^b^: insecticide susceptibility

^c^: insecticide resistance

^d^:Not done

1. **Larvae at Wolgot-myeon in 2023**

| ***#*** | ***Anopheles* spp.** | **Date** | **Place** | **Method of Identification** |
| --- | --- | --- | --- | --- |
|  |  |  |  |  |
| **WG_AL_23_1** | *An. lindesayi* | **2023.09.21-09.22** | **WG** | **Multiplex PCR** |
| **WG_AL_23_2** | *An. lindesayi* | **2023.09.21-09.22** | **WG** | **Multiplex PCR** |
| **WG_AL_23_3** | *An. lindesayi* | **2023.09.21-09.22** | **WG** | **Multiplex PCR** |
| **WG_AL_23_4** | *An. lindesayi* | **2023.09.21-09.22** | **WG** | **Multiplex PCR** |
| **WG_AL_23_5** | *An. lindesayi* | **2023.09.21-09.22** | **WG** | **Multiplex PCR** |
| **WG_AL_23_6** | *An. lindesayi* | **2023.09.21-09.22** | **WG** | **Multiplex PCR** |
| **WG_AL_23_7** | *An. lindesayi* | **2023.09.21-09.22** | **WG** | **Multiplex PCR** |
| **WG_AL_23_8** | *An. lindesayi* | **2023.09.21-09.22** | **WG** | **Multiplex PCR** |
| **WG_AL_23_9** | *An. lindesayi* | **2023.09.21-09.22** | **WG** | **Multiplex PCR** |
| **WG_AL_23_10** | *An. lindesayi* | **2023.09.21-09.22** | **WG** | **Multiplex PCR** |
| **WG_AL_23_11** | *An. lindesayi* | **2023.09.21-09.22** | **WG** | **Multiplex PCR** |
| **WG_AL_23_12** | *An. lindesayi* | **2023.09.21-09.22** | **WG** | **Multiplex PCR** |
| **WG_AL_23_13** | *An. lindesayi* | **2023.09.21-09.22** | **WG** | **Multiplex PCR** |
| **WG_AL_23_14** | *An. lindesayi* | **2023.09.21-09.22** | **WG** | **Multiplex PCR** |
| **WG_AL_23_15** | *An. lindesayi* | **2023.09.21-09.22** | **WG** | **Multiplex PCR** |
| **WG_AL_23_16** | *An. lindesayi* | **2023.09.21-09.22** | **WG** | **Multiplex PCR** |
| **WG_AL_23_17** | *An. lindesayi* | **2023.09.21-09.22** | **WG** | **Multiplex PCR** |
| **WG_AL_23_18** | *An. lindesayi* | **2023.09.21-09.22** | **WG** | **Multiplex PCR** |
| **WG_AL_23_19** | *An. lindesayi* | **2023.09.21-09.22** | **WG** | **Multiplex PCR** |
| **WG_AL_23_20** | *An. lindesayi* | **2023.09.21-09.22** | **WG** | **Multiplex PCR** |
| **WG_AL_23_21** | *An. lindesayi* | **2023.09.21-09.22** | **WG** | **Multiplex PCR** |
| **WG_AL_23_22** | *An. lindesayi* | **2023.09.21-09.22** | **WG** | **Multiplex PCR** |
| **WG_AL_23_23** | *An. lindesayi* | **2023.09.21-09.22** | **WG** | **Multiplex PCR** |
| **WG_AL_23_24** | *An. lindesayi* | **2023.09.21-09.22** | **WG** | **Multiplex PCR** |
| **WG_AL_23_25** | *An. lindesayi* | **2023.09.21-09.22** | **WG** | **Multiplex PCR** |
| **WG_AL_23_26** | *An. lindesayi* | **2023.09.21-09.22** | **WG** | **Multiplex PCR** |
| **WG_AL_23_27** | *An. lindesayi* | **2023.09.21-09.22** | **WG** | **Multiplex PCR** |
| **WG_AL_23_28** | *An. lindesayi* | **2023.09.21-09.22** | **WG** | **Multiplex PCR** |
| **WG_AL_23_29** | *An. lindesayi* | **2023.09.21-09.22** | **WG** | **Multiplex PCR** |
| **WG_AL_23_30** | *An. lindesayi* | **2023.09.21-09.22** | **WG** | **Multiplex PCR** |
| **WG_AL_23_31** | *An. lindesayi* | **2023.09.21-09.22** | **WG** | **Multiplex PCR** |
| **WG_AL_23_32** | *An. lindesayi* | **2023.09.21-09.22** | **WG** | **Multiplex PCR** |
| **WG_AL_23_33** | *An. lindesayi* | **2023.09.21-09.22** | **WG** | **Multiplex PCR** |
| **WG_AL_23_34** | *An. lindesayi* | **2023.09.21-09.22** | **WG** | **Multiplex PCR** |
| **WG_AL_23_35** | *An. lindesayi* | **2023.09.21-09.22** | **WG** | **Multiplex PCR** |
| **WG_AL_23_36** | *An. lindesayi* | **2023.09.21-09.22** | **WG** | **Multiplex PCR** |
| **WG_AL_23_37** | *An. lindesayi* | **2023.09.21-09.22** | **WG** | **Multiplex PCR** |
| **WG_AL_23_38** | *An. lindesayi* | **2023.09.21-09.22** | **WG** | **Multiplex PCR** |
| **WG_AL_23_39** | *An. lindesayi* | **2023.09.21-09.22** | **WG** | **Multiplex PCR** |
| **WG_AL_23_40** | *An. lindesayi* | **2023.09.21-09.22** | **WG** | **Multiplex PCR** |
| **WG_AL_23_41** | *An. lindesayi* | **2023.09.21-09.22** | **WG** | **Multiplex PCR** |
| **WG_AL_23_42** | *An. lindesayi* | **2023.09.21-09.22** | **WG** | **Multiplex PCR** |
| **WG_AL_23_43** | *An. lindesayi* | **2023.09.21-09.22** | **WG** | **Multiplex PCR** |
| **WG_AL_23_44** | *An. lindesayi* | **2023.09.21-09.22** | **WG** | **Multiplex PCR** |
| **WG_AL_23_45** | *An. lindesayi* | **2023.09.21-09.22** | **WG** | **Multiplex PCR** |
| **WG_AL_23_46** | *An. lindesayi* | **2023.09.21-09.22** | **WG** | **Multiplex PCR** |
| **WG_AL_23_47** | *An. lindesayi* | **2023.09.21-09.22** | **WG** | **Multiplex PCR** |
| **WG_AL_23_48** | *An. lindesayi* | **2023.09.21-09.22** | **WG** | **Multiplex PCR** |
| **WG_AL_23_49** | *An. lindesayi* | **2023.09.21-09.22** | **WG** | **Multiplex PCR** |
| **WG_AL_23_50** | *An. lindesayi* | **2023.09.21-09.22** | **WG** | **Multiplex PCR** |
| **WG_AL_23_51** | *An. lindesayi* | **2023.09.21-09.22** | **WG** | **Multiplex PCR** |

1. **Adult at Wolgot-myeon in 2023**

| ***#*** | ***Anopheles* spp.** | **Date** | **Place** | **Method of Identification** |
| --- | --- | --- | --- | --- |
|  |  |  |  |  |
| **WG_AA_23_1** | ***An. lindesayi*** | **2023.07.01-07.02** | **WG** | **Multiplex PCR** |
| **WG_AA_23_2** | ***An. lindesayi*** | **2023.07.01-07.02** | **WG** | **Multiplex PCR** |
| **WG_AA_23_3** | ***An. lindesayi*** | **2023.07.01-07.02** | **WG** | **Multiplex PCR** |
| **WG_AA_23_4** | ***An. lindesayi*** | **2023.07.01-07.02** | **WG** | **Multiplex PCR** |
| **WG_AA_23_5** | ***An. lindesayi*** | **2023.07.01-07.02** | **WG** | **Multiplex PCR** |
| **WG_AA_23_6** | ***An. lindesayi*** | **2023.07.01-07.02** | **WG** | **Multiplex PCR** |
| **WG_AA_23_7** | ***An. lindesayi*** | **2023.07.01-07.02** | **WG** | **Multiplex PCR** |
| **WG_AA_23_8** | ***An. lindesayi*** | **2023.07.01-07.02** | **WG** | **Multiplex PCR** |
| **WG_AA_23_9** | ***An. lindesayi*** | **2023.07.01-07.02** | **WG** | **Multiplex PCR** |
| **WG_AA_23_10** | ***An. lindesayi*** | **2023.07.01-07.02** | **WG** | **Multiplex PCR** |
| **WG_AA_23_11** | ***An. lindesayi*** | **2023.07.01-07.02** | **WG** | **Multiplex PCR** |
| **WG_AA_23_12** | ***An. lindesayi*** | **2023.07.01-07.02** | **WG** | **Multiplex PCR** |
| **WG_AA_23_13** | ***An. lindesayi*** | **2023.07.01-07.02** | **WG** | **Multiplex PCR** |
| **WG_AA_23_14** | ***An. lindesayi*** | **2023.07.01-07.02** | **WG** | **Multiplex PCR** |
| **WG_AA_23_15** | ***An. lindesayi*** | **2023.07.01-07.02** | **WG** | **Multiplex PCR** |
| **WG_AA_23_16** | ***An. lindesayi*** | **2023.07.01-07.02** | **WG** | **Multiplex PCR** |
| **WG_AA_23_17** | ***An. kleini*** | **2023.07.01-07.02** | **WG** | **Multiplex PCR** |
| **WG_AA_23_18** | ***An. kleini*** | **2023.07.01-07.02** | **WG** | **Multiplex PCR** |
| **WG_AA_23_19** | ***An. sinensis*** | **2023.08.16-08.17** | **WG** | **Multiplex PCR** |
| **WG_AA_23_20** | ***An. sinensis*** | **2023.08.16-08.17** | **WG** | **Multiplex PCR** |
| **WG_AA_23_21** | ***An. sinensis*** | **2023.08.16-08.17** | **WG** | **Multiplex PCR** |

1. **Larvae at Naega-myeon in 2023**

| ***#*** | ***Anopheles* spp.** | **Date** | **Place** | **Method of Identification** | ***kdr* type** | **susceptibility (S) ^b^ or resistance (R) ^c^** |
| --- | --- | --- | --- | --- | --- | --- |
|  |  |  |  |  |  |  |
| **NG_AL_23_1** | ***An. sineroides*** | **2023.10.05** | **NG** | **ITS2** | **TTA** | **(S)** |
| **NG_AL_23_2** | ***An. sineroides*** | **2023.10.05** | **NG** | **ITS2** | **TTA** | **(S)** |
| **NG_AL_23_3** | ***An. sineroides*** | **2023.10.05** | **NG** | **ITS2** | **TTA** | **(S)** |
| **NG_AL_23_4** | ***An. sineroides*** | **2023.10.05** | **NG** | **ITS2** | **ND** | **ND** |
| **NG_AL_23_5** | ***An. sineroides*** | **2023.10.05** | **NG** | **ITS2** | **ND** | **ND** |
| **NG_AL_23_6** | ***An. sineroides*** | **2023.10.05** | **NG** | **ITS2** | **ND** | **ND** |
| **NG_AL_23_7** | ***An. sineroides*** | **2023.10.05** | **NG** | **ITS2** | **ND** | **ND** |
| **NG_AL_23_8** | ***An. sineroides*** | **2023.10.05** | **NG** | **ITS2** | **ND** | **ND** |
| **NG_AL_23_9** | ***An. sineroides*** | **2023.10.05** | **NG** | **ITS2** | **ND** | **ND** |
| **NG_AL_23_10** | ***An. sineroides*** | **2023.10.05** | **NG** | **ITS2** | **ND** | **ND** |
| **NG_AL_23_11** | ***An. sineroides*** | **2023.10.05** | **NG** | **ITS2** | **ND** | **ND** |
| **NG_AL_23_12** | ***An. sineroides*** | **2023.10.05** | **NG** | **ITS2** | **ND** | **ND** |
| **NG_AL_23_13** | ***An. sineroides*** | **2023.10.05** | **NG** | **ITS2** | **ND** | **ND** |
| **NG_AL_23_14** | ***An. sineroides*** | **2023.10.05** | **NG** | **ITS2** | **ND** | **ND** |
| **NG_AL_23_15** | ***An. sineroides*** | **2023.10.05** | **NG** | **ITS2** | **ND** | **ND** |
| **NG_AL_23_16** | ***An. sineroides*** | **2023.10.05** | **NG** | **ITS2** | **ND** | **ND** |
| **NG_AL_23_17** | ***An. sineroides*** | **2023.10.05** | **NG** | **ITS2** | **ND** | **ND** |
| **NG_AL_23_18** | ***An. sineroides*** | **2023.10.05** | **NG** | **ITS2** | **ND** | **ND** |
| **NG_AL_23_19** | ***An. sineroides*** | **2023.10.05** | **NG** | **ITS2** | **ND** | **ND** |
| **NG_AL_23_20** | ***An. sineroides*** | **2023.10.05** | **NG** | **ITS2** | **ND** | **ND** |
| **NG_AL_23_21** | ***An. sineroides*** | **2023.10.05** | **NG** | **ITS2** | **ND** | **ND** |
| **NG_AL_23_22** | ***An. sineroides*** | **2023.10.05** | **NG** | **ITS2** | **ND** | **ND** |
| **NG_AL_23_23** | ***An. sineroides*** | **2023.10.05** | **NG** | **ITS2** | **ND** | **ND** |
| **NG_AL_23_24** | ***An. sineroides*** | **2023.10.05** | **NG** | **ITS2** | **ND** | **ND** |
| **NG_AL_23_25** | ***An. sineroides*** | **2023.10.05** | **NG** | **ITS2** | **ND** | **ND** |
| **NG_AL_23_26** | ***An. sineroides*** | **2023.10.05** | **NG** | **ITS2** | **ND** | **ND** |
| **NG_AL_23_27** | ***An. sineroides*** | **2023.10.05** | **NG** | **ITS2** | **ND** | **ND** |
| **NG_AL_23_28** | ***An. sineroides*** | **2023.10.05** | **NG** | **ITS2** | **ND** | **ND** |
| **NG_AL_23_29** | ***An. sineroides*** | **2023.10.05** | **NG** | **ITS2** | **ND** | **ND** |
| **NG_AL_23_30** | ***An. sineroides*** | **2023.10.05** | **NG** | **ITS2** | **ND** | **ND** |
| **NG_AL_23_31** | ***An. sineroides*** | **2023.10.05** | **NG** | **ITS2** | **ND** | **ND** |
| **NG_AL_23_32** | ***An. sineroides*** | **2023.10.05** | **NG** | **ITS2** | **ND** | **ND** |
| **NG_AL_23_33** | ***An. sineroides*** | **2023.10.05** | **NG** | **ITS2** | **ND** | **ND** |
| **NG_AL_23_34** | ***An. sineroides*** | **2023.10.05** | **NG** | **ITS2** | **ND** | **ND** |
| **NG_AL_23_35** | ***An. sineroides*** | **2023.10.05** | **NG** | **ITS2** | **ND** | **ND** |
| **NG_AL_23_36** | ***An. sineroides*** | **2023.10.05** | **NG** | **ITS2** | **ND** | **ND** |
| **NG_AL_23_37** | ***An. sineroides*** | **2023.10.05** | **NG** | **ITS2** | **ND** | **ND** |
| **NG_AL_23_38** | ***An. sineroides*** | **2023.10.05** | **NG** | **ITS2** | **ND** | **ND** |
| **NG_AL_23_39** | ***An. sineroides*** | **2023.10.05** | **NG** | **ITS2** | **ND** | **ND** |
| **NG_AL_23_40** | ***An. sineroides*** | **2023.10.05** | **NG** | **ITS2** | **ND** | **ND** |
| **NG_AL_23_41** | ***An. sineroides*** | **2023.10.05** | **NG** | **ITS2** | **ND** | **ND** |
| **NG_AL_23_42** | ***An. sineroides*** | **2023.10.05** | **NG** | **ITS2** | **ND** | **ND** |
| **NG_AL_23_43** | ***An. sineroides*** | **2023.10.05** | **NG** | **ITS2** | **ND** | **ND** |
| **NG_AL_23_44** | ***An. sineroides*** | **2023.10.05** | **NG** | **ITS2** | **ND** | **ND** |
| **NG_AL_23_45** | ***An. sineroides*** | **2023.10.05** | **NG** | **ITS2** | **ND** | **ND** |
| **NG_AL_23_46** | ***An. sineroides*** | **2023.10.05** | **NG** | **ITS2** | **ND** | **ND** |
| **NG_AL_23_47** | ***An. sineroides*** | **2023.10.05** | **NG** | **ITS2** | **ND** | **ND** |
| **NG_AL_23_48** | ***An. sineroides*** | **2023.10.05** | **NG** | **ITS2** | **ND** | **ND** |
| **NG_AL_23_49** | ***An. sineroides*** | **2023.10.05** | **NG** | **ITS2** | **ND** | **ND** |
| **NG_AL_23_50** | ***An. sineroides*** | **2023.10.05** | **NG** | **ITS2** | **ND** | **ND** |
| **NG_AL_23_51** | ***An. sineroides*** | **2023.10.05** | **NG** | **ITS2** | **ND** | **ND** |
| **NG_AL_23_52** | ***An. sineroides*** | **2023.10.05** | **NG** | **ITS2** | **ND** | **ND** |
| **NG_AL_23_53** | ***An. sineroides*** | **2023.10.05** | **NG** | **ITS2** | **ND** | **ND** |
| **NG_AL_23_54** | ***An. sineroides*** | **2023.10.05** | **NG** | **ITS2** | **ND** | **ND** |
| **NG_AL_23_55** | ***An. sineroides*** | **2023.10.05** | **NG** | **ITS2** | **ND** | **ND** |

1. **Adults at Naega-myeon in 2023**

| ***#*** | ***Anopheles* spp.** | **Date** | **Place** | **Method of Identification** | ***kdr* type** | **susceptibility (S) ^b^ or resistance (R) ^c^** |
| --- | --- | --- | --- | --- | --- | --- |
|  |  |  |  |  |  |  |
| **NG_AA_23_1** | ***An. sinensis*** | **2023.08.01-08.02** | **NG** | **Multiplex PCR, ITS2, COI** | **TTT(F)/TTT(F)** | **R** |
| **NG_AA_23_2** | ***An. sinensis*** | **2023.08.01-08.02** | **NG** | **Multiplex PCR, COI** | **TTT(F)/TGT(C)** | **R** |
| **NG_AA_23_3** | ***An. sinensis*** | **2023.08.01-08.02** | **NG** | **Multiplex PCR, ITS2** | **TTG(L)/TTT(F)** | **R** |
| **NG_AA_23_4** | ***An. sinensis*** | **2023.08.01-08.02** | **NG** | **Multiplex PCR, ITS2, COI** | **TTG(L)/TTT(F)** | **R** |
| **NG_AA_23_5** | ***An. sinensis*** | **2023.08.01-08.02** | **NG** | **Multiplex PCR, COI** | **TTG(L)/TTT(F)** | **R** |
| **NG_AA_23_6** | ***An. sinensis*** | **2023.08.01-08.02** | **NG** | **Multiplex PCR, COI** | **TTG(L)/TTT(F)** | **R** |
| **NG_AA_23_7** | ***An. sinensis*** | **2023.08.01-08.02** | **NG** | **Multiplex PCR, COI** | **TTG(L)/TTT(F)** | **R** |
| **NG_AA_23_8** | ***An. sinensis*** | **2023.08.01-08.02** | **NG** | **Multiplex PCR, COI** | **TTG(L)/TTT(F)** | **R** |
| **NG_AA_23_9** | ***An. sinensis*** | **2023.08.01-08.02** | **NG** | **Multiplex PCR, ITS2** | **ND** | **ND** |
| **NG_AA_23_10** | ***An. sinensis*** | **2023.08.01-08.02** | **NG** | **Multiplex PCR, ITS2** | **ND** | **ND** |
| **NG_AA_23_11** | ***An. sinensis*** | **2023.08.01-08.02** | **NG** | **Multiplex PCR** | **ND** | **ND** |
| **NG_AA_23_12** | ***An. sinensis*** | **2023.08.01-08.02** | **NG** | **Multiplex PCR** | **ND** | **ND** |
| **NG_AA_23_13** | ***An. sinensis*** | **2023.08.16-08.17** | **NG** | **Multiplex PCR, ITS2, COI** | **TTG(L)/TTT(F)** | **R** |
| **NG_AA_23_14** | ***An. sinensis*** | **2023.08.16-08.17** | **NG** | **Multiplex PCR, ITS2, COI** | **TTG(L)/TTC^a^(F)** | **R** |
| **NG_AA_23_15** | ***An. sinensis*** | **2023.08.16-08.17** | **NG** | **Multiplex PCR** | **TTG(L)/TGT(C)** | **R** |
| **NG_AA_23_16** | ***An. sinensis*** | **2023.08.16-08.17** | **NG** | **Multiplex PCR** | **TTG(L)/TTT(F)** | **R** |
| **NG_AA_23_17** | ***An. sinensis*** | **2023.08.16-08.17** | **NG** | **Multiplex PCR, COI** | **TTG(L)/TTT(F)** | **R** |
| **NG_AA_23_18** | ***An. sinensis*** | **2023.08.16-08.17** | **NG** | **Multiplex PCR** | **ND** | **ND** |
| **NG_AA_23_19** | ***An. sinensis*** | **2023.08.16-08.17** | **NG** | **Multiplex PCR** | **ND** | **ND** |
| **NG_AA_23_20** | ***An. sinensis*** | **2023.08.16-08.17** | **NG** | **Multiplex PCR** | **ND** | **ND** |
| **NG_AA_23_21** | ***An. sinensis*** | **2023.08.16-08.17** | **NG** | **Multiplex PCR, ITS2, COI** | **TTG(L)/TTT(F)** | **R** |
| **NG_AA_23_22** | ***An. sinensis*** | **2023.08.16-08.17** | **NG** | **Multiplex PCR, ITS2, COI** | **TTG(L)/TTT(F)** | **R** |
| **NG_AA_23_23** | ***An. sinensis*** | **2023.08.16-08.17** | **NG** | **Multiplex PCR, ITS2, COI** | **TTG(L)/TGT(C)** | **R** |
| **NG_AA_23_24** | ***An. sinensis*** | **2023.08.16-08.17** | **NG** | **Multiplex PCR, ITS2, COI** | **TTG(L)/TTT(F)** | **R** |
| **NG_AA_23_25** | ***An. sinensis*** | **2023.08.16-08.17** | **NG** | **Multiplex PCR, ITS2, COI** | **ND** | **ND** |
| **NG_AA_23_26** | ***An. sinensis*** | **2023.08.16-08.17** | **NG** | **Multiplex PCR, ITS2** | **TTT(F)/TTT(F)** | **R** |
| **NG_AA_23_27** | ***An. sinensis*** | **2023.08.16-08.17** | **NG** | **Multiplex PCR** | **ND** | **ND** |
| **NG_AA_23_28** | ***An. sinensis*** | **2023.08.16-08.17** | **NG** | **Multiplex PCR** | **ND** | **ND** |
| **NG_AA_23_29** | ***An. sinensis*** | **2023.08.16-08.17** | **NG** | **Multiplex PCR, ITS2, COI** | **TTG(L)/TGT(C)** | **R** |
| **NG_AA_23_30** | ***An. sinensis*** | **2023.08.16-08.17** | **NG** | **Multiplex PCR, ITS2, COI** | **TTG(L)/TTG(L)** | **S** |
| **NG_AA_23_31** | ***An. sinensis*** | **2023.08.16-08.17** | **NG** | **Multiplex PCR, ITS2, COI** | **TGT(C)/TGT(C)** | **R** |
| **NG_AA_23_32** | ***An. sinensis*** | **2023.08.16-08.17** | **NG** | **Multiplex PCR** | **ND** | **ND** |
| **NG_AA_23_33** | ***An. sinensis*** | **2023.08.16-08.17** | **NG** | **Multiplex PCR, ITS2, COI** | **TTT(F)/TTT(F)** | **R** |
| **NG_AA_23_34** | ***An. sineroides*** | **2023.08.16-08.17** | **NG** | **Multiplex PCR, ITS2** | **ND** | **ND** |
| **NG_AA_23_35** | ***An. belenrae*** | **2023.08.16-08.17** | **NG** | **Multiplex PCR** | **ND** | **ND** |
| **NG_AA_23_36** | ***An. sinensis*** | **2023.09.11-09.12** | **NG** | **Multiplex PCR, COI** | **TTG(L)/TTT(F)** | **R** |
| **NG_AA_23_37** | ***An. sinensis*** | **2023.09.11-09.12** | **NG** | **Multiplex PCR, COI** | **TTG(L)/TTT(F)** | **R** |
| **NG_AA_23_38** | ***An. sinensis*** | **2023.09.11-09.12** | **NG** | **Multiplex PCR** | **TGT(C)/TGT (C)** | **R** |
| **NG_AA_23_39** | ***An. sinensis*** | **2023.09.11-09.12** | **NG** | **Multiplex PCR, ITS2, COI** | **TTG(L)/TGT(C)** | **R** |
| **NG_AA_23_40** | ***An. sinensis*** | **2023.09.11-09.12** | **NG** | **Multiplex PCR, COI** | **ND** | **ND** |
| **NG_AA_23_41** | ***An. sinensis*** | **2023.09.11-09.12** | **NG** | **Multiplex PCR, ITS2, COI** | **TTG(L)/TTT(F)** | **R** |
| **NG_AA_23_42** | ***An. sinensis*** | **2023.09.11-09.12** | **NG** | **Multiplex PCR, ITS2, COI** | **TGT(C)/TGT(C)** | **R** |
| **NG_AA_23_43** | ***An. sinensis*** | **2023.09.11-09.12** | **NG** | **Multiplex PCR, ITS2, COI** | **TTT(F)/TGT(C)** | **R** |
| **NG_AA_23_44** | ***An. sinensis*** | **2023.09.11-09.12** | **NG** | **Multiplex PCR, ITS2, COI** | **TTG(L)/TTT(F)** | **R** |
| **NG_AA_23_45** | ***An. sinensis*** | **2023.09.11-09.12** | **NG** | **Multiplex PCR, ITS2, COI** | **TTG(L)/TTT(F)** | **R** |
| **NG_AA_23_46** | ***An. sinensis*** | **2023.09.11-09.12** | **NG** | **Multiplex PCR, ITS2, COI** | **TTG(L)/TTT(F)** | **R** |
| **NG_AA_23_47** | ***An. sinensis*** | **2023.09.11-09.12** | **NG** | **Multiplex PCR, ITS2, COI** | **TTG(L)/TTT(F)** | **R** |
| **NG_AA_23_48** | ***An. sinensis*** | **2023.09.11-09.12** | **NG** | **Multiplex PCR, ITS2, COI** | **TGT(C)/TGT(C)** | **R** |
| **NG_AA_23_49** | ***An. sinensis*** | **2023.09.11-09.12** | **NG** | **Multiplex PCR, ITS2, COI** | **TTG(L)/TTT(F)** | **R** |
| **NG_AA_23_50** | ***An. sinensis*** | **2023.09.11-09.12** | **NG** | **Multiplex PCR, ITS2, COI** | **TTG(L)/TTT(F)** | **R** |
| **NG_AA_23_51** | ***An. sinensis*** | **2023.09.11-09.12** | **NG** | **Multiplex PCR** | **ND** | **ND** |
| **NG_AA_23_52** | ***An. sinensis*** | **2023.09.11-09.12** | **NG** | **Multiplex PCR** | **ND** | **ND** |
| **NG_AA_23_53** | ***An. sinensis*** | **2023.09.11-09.12** | **NG** | **Multiplex PCR** | **TGT(C)/TGT(C)** | **R** |
| **NG_AA_23_54** | ***An. sinensis*** | **2023.09.11-09.12** | **NG** | **Multiplex PCR, ITS2, COI** | **ND** | **ND** |
| **NG_AA_23_55** | ***An. sinensis*** | **2023.09.11-09.12** | **NG** | **Multiplex PCR** | **TTG(L)/TGT(C)** | **R** |
| **NG_AA_23_56** | ***An. sinensis*** | **2023.09.11-09.12** | **NG** | **Multiplex PCR, COI** | **TTT(F)/TGT(C)** | **R** |
| **NG_AA_23_57** | ***An. sinensis*** | **2023.09.11-09.12** | **NG** | **Multiplex PCR, ITS2, COI** | **TTG(L)/TTT(F)** | **R** |
| **NG_AA_23_58** | ***An. sinensis*** | **2023.09.11-09.12** | **NG** | **Multiplex PCR** | **TGT(C)/TGT(C)** | **R** |
| **NG_AA_23_59** | ***An. sinensis*** | **2023.09.11-09.12** | **NG** | **Multiplex PCR, ITS2, COI** | **TTT(F)/TGT(C)** | **R** |
| **NG_AA_23_60** | ***An. sinensis*** | **2023.09.11-09.12** | **NG** | **Multiplex PCR** | **TTG(L)/TTT(F)** | **R** |
| **NG_AA_23_61** | ***An. sinensis*** | **2023.09.11-09.12** | **NG** | **Multiplex PCR** | **TTG(L)/TTT(F)** | **R** |
| **NG_AA_23_62** | ***An. sinensis*** | **2023.09.11-09.12** | **NG** | **Multiplex PCR** | **TTG(L)/TTT(F)** | **R** |
| **NG_AA_23_63** | ***An. sinensis*** | **2023.09.11-09.12** | **NG** | **Multiplex PCR** | **TTG(L)/TTT(F)** | **R** |
| **NG_AA_23_64** | ***An. sinensis*** | **2023.09.11-09.12** | **NG** | **Multiplex PCR** | **TGT(C)/TGT(C)** | **R** |
| **NG_AA_23_65** | ***An. sinensis*** | **2023.09.11-09.12** | **NG** | **Multiplex PCR, ITS2, COI** | **TTG(L)/TTT(F)** | **R** |
| **NG_AA_23_66** | ***An. sinensis*** | **2023.09.11-09.12** | **NG** | **Multiplex PCR** | **ND** | **ND** |
| **NG_AA_23_67** | ***An. sinensis*** | **2023.09.11-09.12** | **NG** | **Multiplex PCR, ITS2, COI** | **ND** | **ND** |
| **NG_AA_23_68** | ***An. sinensis*** | **2023.09.11-09.12** | **NG** | **Multiplex PCR, ITS2, COI** | **ND** | **ND** |
| **NG_AA_23_69** | ***An. sinensis*** | **2023.09.11-09.12** | **NG** | **Multiplex PCR, ITS2, COI** | **ND** | **ND** |
| **NG_AA_23_70** | ***An. sinensis*** | **2023.09.11-09.12** | **NG** | **Multiplex PCR, ITS2, COI** | **ND** | **ND** |
| **NG_AA_23_71** | ***An. sinensis*** | **2023.09.11-09.12** | **NG** | **Multiplex PCR, ITS2, COI** | **ND** | **ND** |
| **NG_AA_23_72** | ***An. sinensis*** | **2023.09.11-09.12** | **NG** | **Multiplex PCR, ITS2, COI** | **ND** | **ND** |
| **NG_AA_23_73** | ***An. sinensis*** | **2023.09.11-09.12** | **NG** | **Multiplex PCR, ITS2, COI** | **ND** | **ND** |
| **NG_AA_23_74** | ***An. sinensis*** | **2023.09.11-09.12** | **NG** | **Multiplex PCR, ITS2, COI** | **ND** | **ND** |
| **NG_AA_23_75** | ***An. sinensis*** | **2023.09.11-09.12** | **NG** | **Multiplex PCR** | **TGT(C)/TGT(C)** | **R** |
| **NG_AA_23_76** | ***An. sinensis*** | **2023.09.11-09.12** | **NG** | **Multiplex PCR, ITS2** | **TTG(L)/TTT(F)** | **R** |
| **NG_AA_23_77** | ***An. sinensis*** | **2023.09.11-09.12** | **NG** | **Multiplex PCR** | **TTT(F)/TTT(F)** | **R** |
| **NG_AA_23_78** | ***An. sinensis*** | **2023.09.11-09.12** | **NG** | **Multiplex PCR, ITS2, COI** | **TTG(L)/TTG(L)** | **S** |
| **NG_AA_23_79** | ***An. sinensis*** | **2023.09.11-09.12** | **NG** | **Multiplex PCR, ITS2** | **TTG(L)/TGT(C)** | **R** |
| **NG_AA_23_80** | ***An. sinensis*** | **2023.09.11-09.12** | **NG** | **Multiplex PCR, ITS2** | **TTG(L)/TGT(C)** | **R** |
| **NG_AA_23_81** | ***An. sinensis*** | **2023.09.11-09.12** | **NG** | **Multiplex PCR, ITS2, COI** | **TTG(L)/TGT(C)** | **R** |
| **NG_AA_23_82** | ***An. sinensis*** | **2023.09.11-09.12** | **NG** | **Multiplex PCR, ITS2, COI** | **TTG(L)/TTT(F)** | **R** |
| **NG_AA_23_83** | ***An. sinensis*** | **2023.09.11-09.12** | **NG** | **Multiplex PCR, ITS2, COI** | **ND** | **ND** |
| **NG_AA_23_84** | ***An. sinensis*** | **2023.09.11-09.12** | **NG** | **Multiplex PCR, ITS2, COI** | **TTT(F)/TGT(C)** | **R** |
| **NG_AA_23_85** | ***An. sinensis*** | **2023.09.11-09.12** | **NG** | **Multiplex PCR, ITS2, COI** | **ND** | **ND** |
| **NG_AA_23_86** | ***An. sinensis*** | **2023.09.11-09.12** | **NG** | **Multiplex PCR, ITS2, COI** | **ND** | **ND** |
| **NG_AA_23_87** | ***An. sinensis*** | **2023.09.11-09.12** | **NG** | **Multiplex PCR, ITS2, COI** | **ND** | **ND** |
| **NG_AA_23_88** | ***An. sinensis*** | **2023.09.11-09.12** | **NG** | **Multiplex PCR, ITS2, COI** | **TTG(L)/TTT(F)** | **R** |
| **NG_AA_23_89** | ***An. sinensis*** | **2023.09.11-09.12** | **NG** | **Multiplex PCR** | **TTG(L)/TGT(C)** | **R** |
| **NG_AA_23_90** | ***An. sinensis*** | **2023.09.11-09.12** | **NG** | **Multiplex PCR** | **TGT(C)/TGT(C)** | **R** |
| **NG_AA_23_91** | ***An. sinensis*** | **2023.09.11-09.13** | **NG** | **Multiplex PCR, ITS2** | **TTG(L)/TGT(C)** | **R** |
| **NG_AA_23_92** | ***An. pullus*** | **2023.09.11-09.12** | **NG** | **Multiplex PCR** | **ND** | **ND** |
| **NG_AA_23_93** | ***An. belenrae*** | **2023.09.11-09.12** | **NG** | **Multiplex PCR** | **ND** | **ND** |
| **NG_AA_23_94** | ***An. sineroides*** | **2023.09.21-09.22** | **NG** | **Multiplex PCR** | **ND** | **ND** |

^a^: TTG(L)/TTC(F) in L1014F kdr


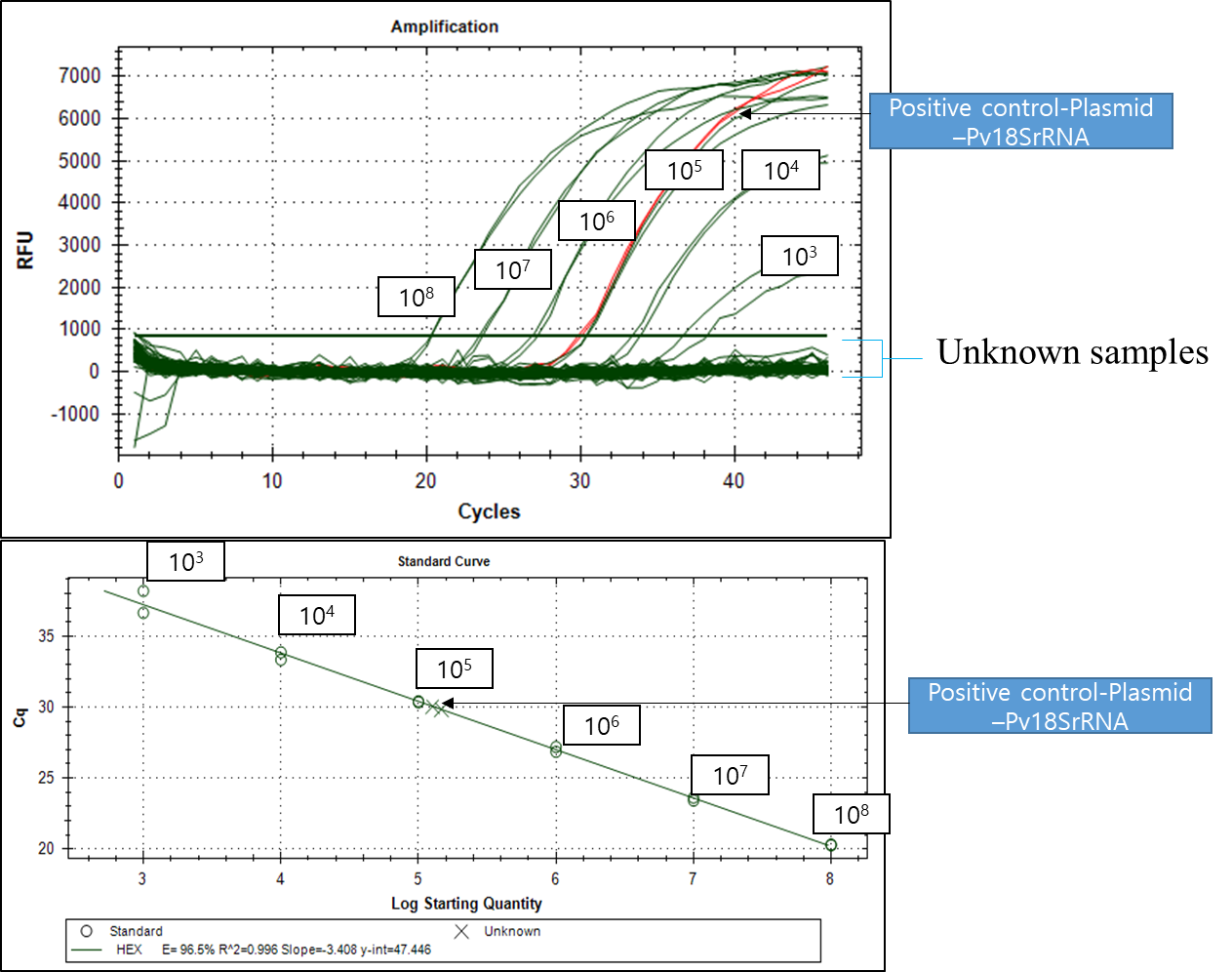


**Fig. S3. RT-PCR result of *Anopheles* adults for detection of *Plasmodium vivax.***


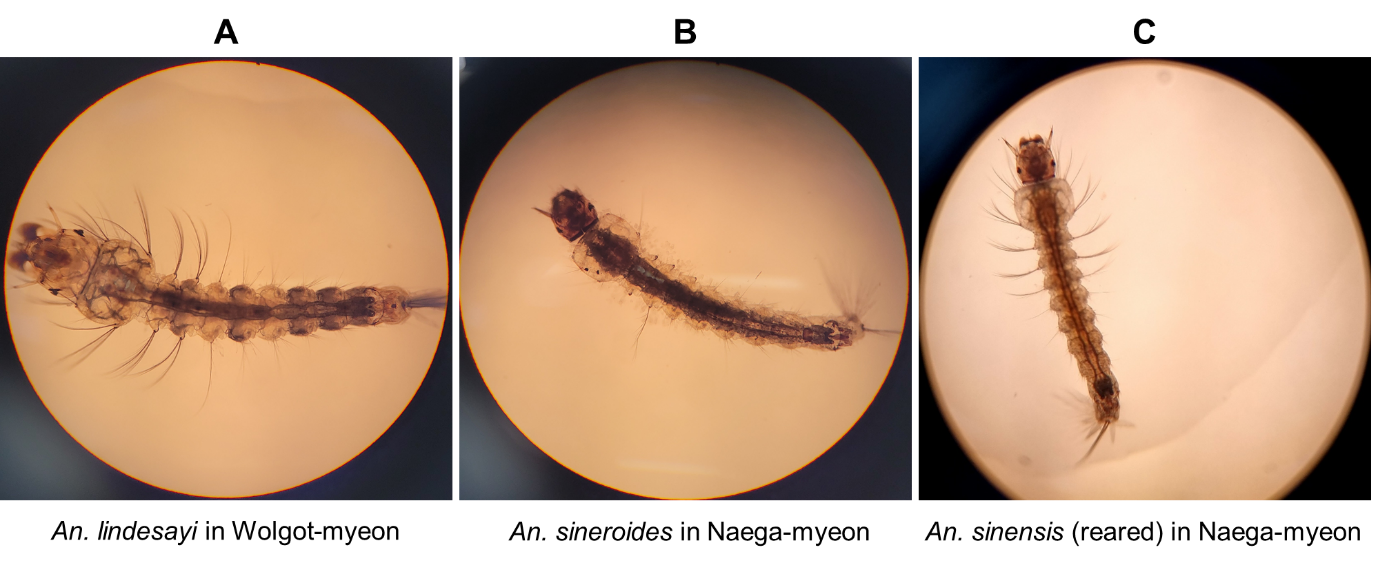


**Fig. S4. Morphology of the *Anopheles* spp. larvae in two breeding sites in 2023.**

**
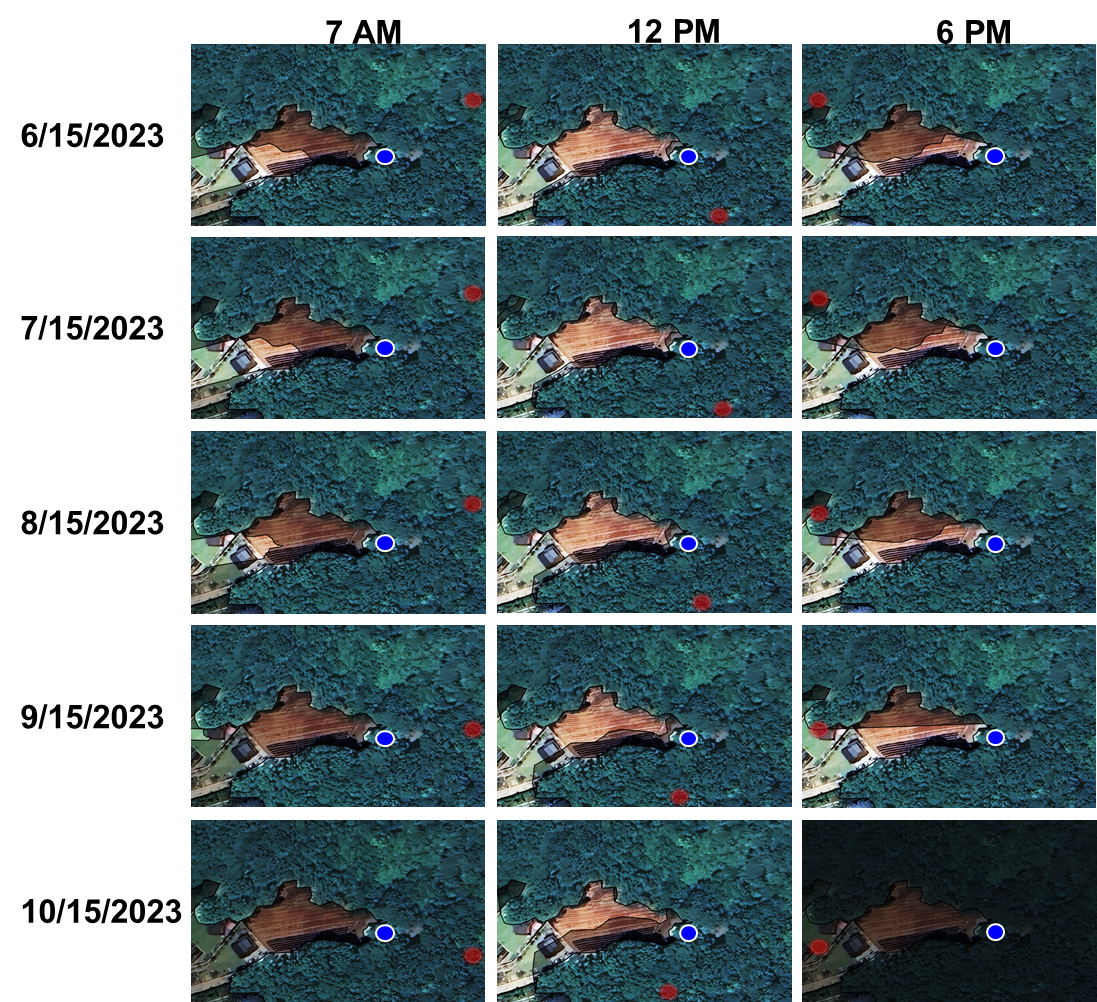
**

**Fig. S5. Time-series of shade of sun light from June to October in breeding site of Wolgot-myeon.**


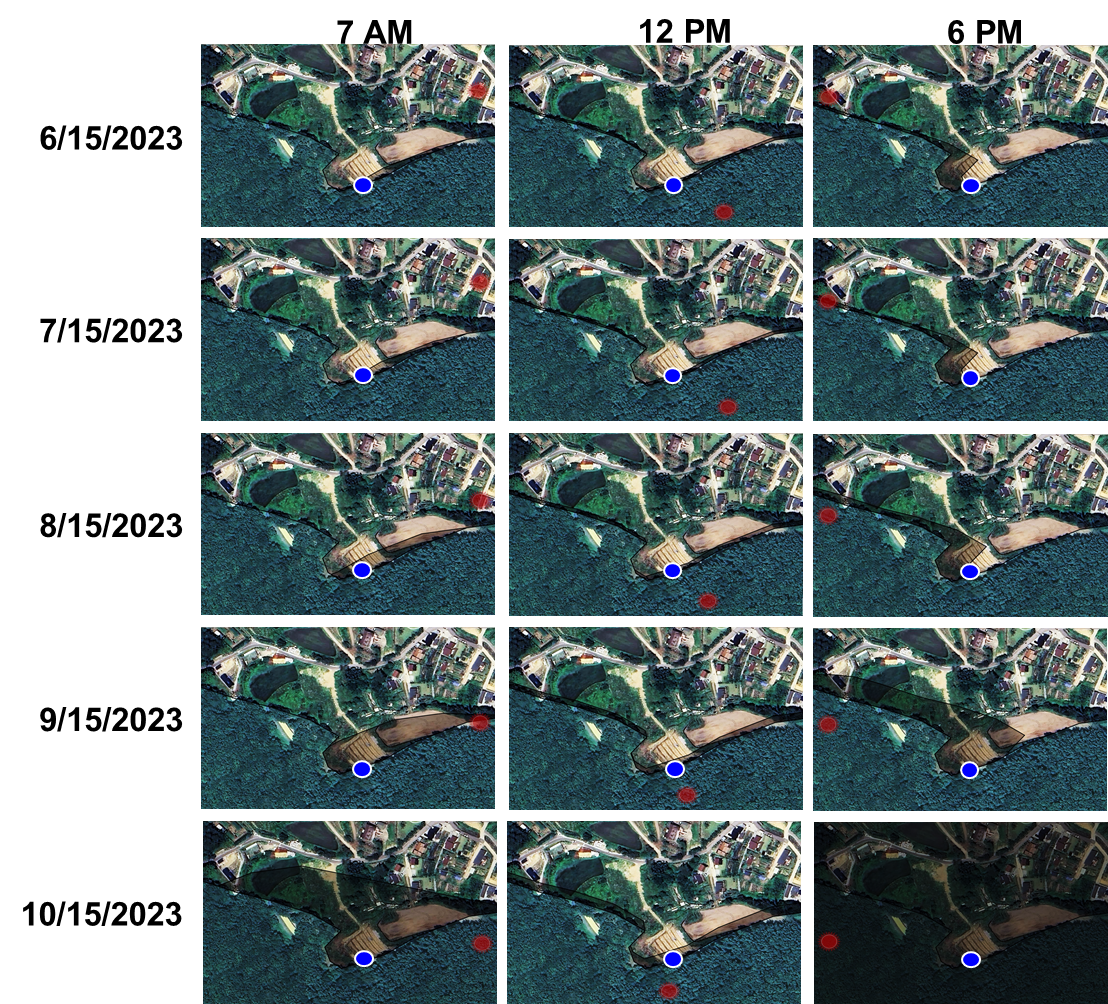


**Fig. S6. Time-series of shade of sun light from June to October in breeding site of Naega-myeon.**

**
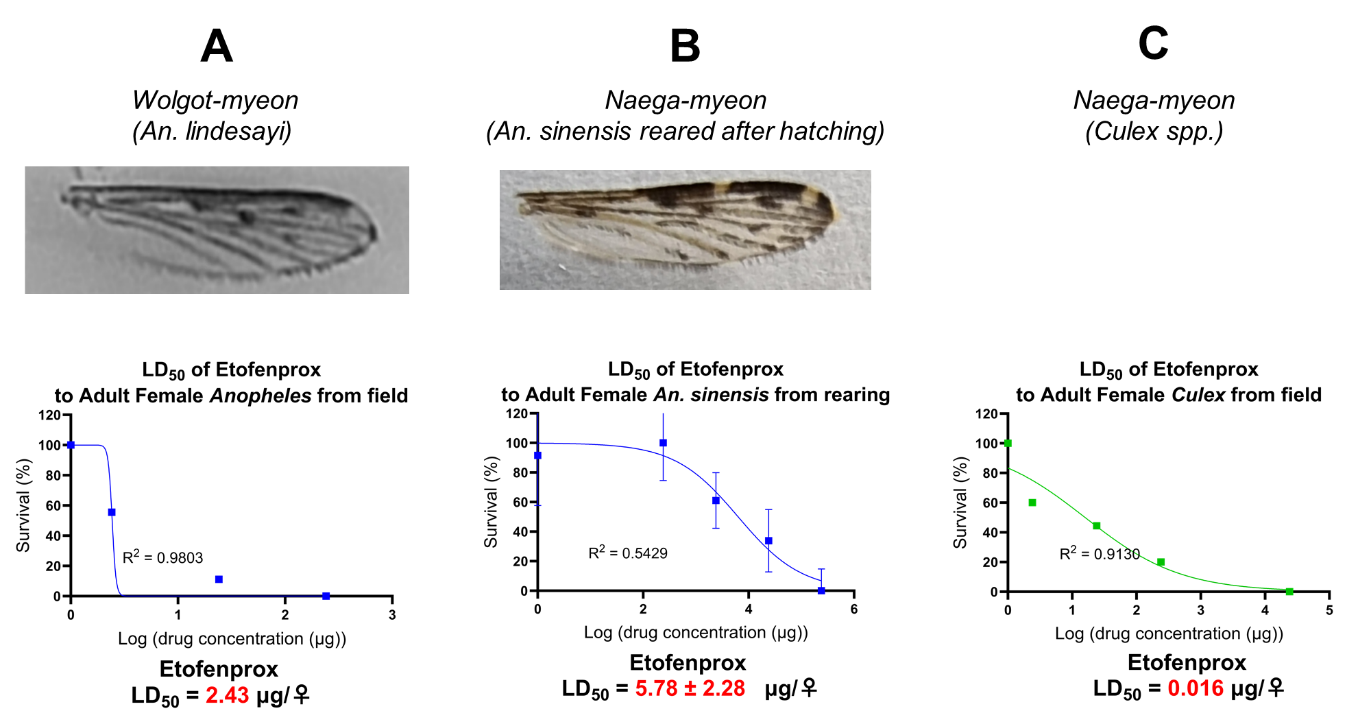
**

**Fig. S7. Toxicities of insecticide (Etofenprox of Pyrethroid group) to adults of *Anopheles* spp..** They were tested with adults derived from field or reared condition.

**
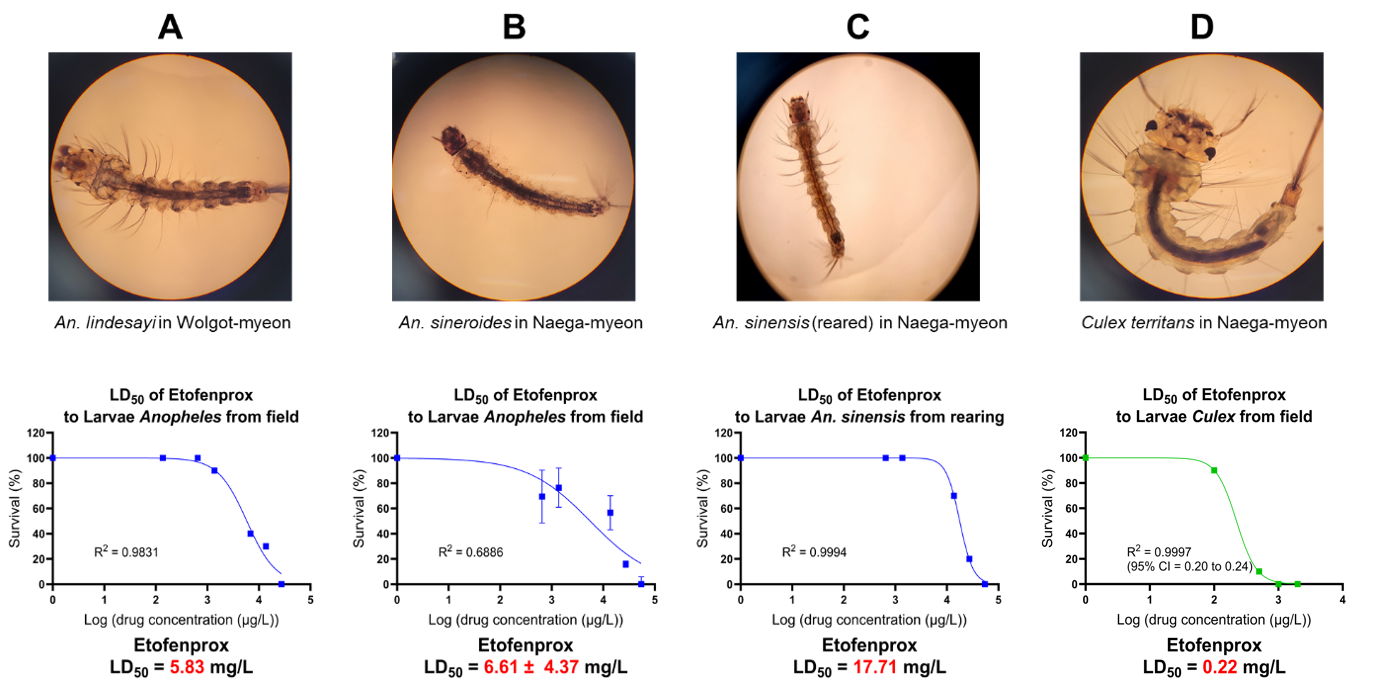
**

**Fig. S8. Toxicities of insecticide (Etofenprox of Pyrethroid group) to larvae of *Anopheles* spp.**

**Table S2. Raw *kdr* gene sequence with mutant genotype (L1014F) of *An. belenrae* (GenBank accession #: OQ303977)**

| ***ITS2*** | GenBank: OQ303977 |
| --- | --- |
| ***kdr*** | GenBank: OQ723439 |
|  | Date of isolation: 2022.09.20-21 |
|  | Place of isolation: Gimpo, Gyeonggi (37°745’–37°750’ N, 126°532’–126°535’ E) |
|  | *AAGCAGTTTGCGTGCTGTGCGGGGAGTGGATCGAATCAATGTGGGACTGTATGCTAGTCGGGGATGTGTCATGCATCCCATTCTTCTTAGCCACTGTGGTAATTGGAAACTTGGTGGTGAGTAACTGCAGGACGACAGGTCAGCACTTTCTCCGATTCTAACAAGACGCTTTGGTTGCAGGTACTTAATCTTTTCTTAGCTTTGCTTTTGTCCAATTTCGGATCATCATCGCTCTCTGCACCAACGGCGGATAACGAGACGAATAAAATCGCTGAAGCGTTCAACCGAATTTCCCGCTTCTCTAACTGGATCAAAATGAACGTAGCAAATGCGCTAAC* |
|  | Genotype: Heterozygous resistant genotype with **TTG(L)/TTT(F)** |
|  | *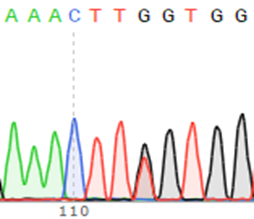* |

**
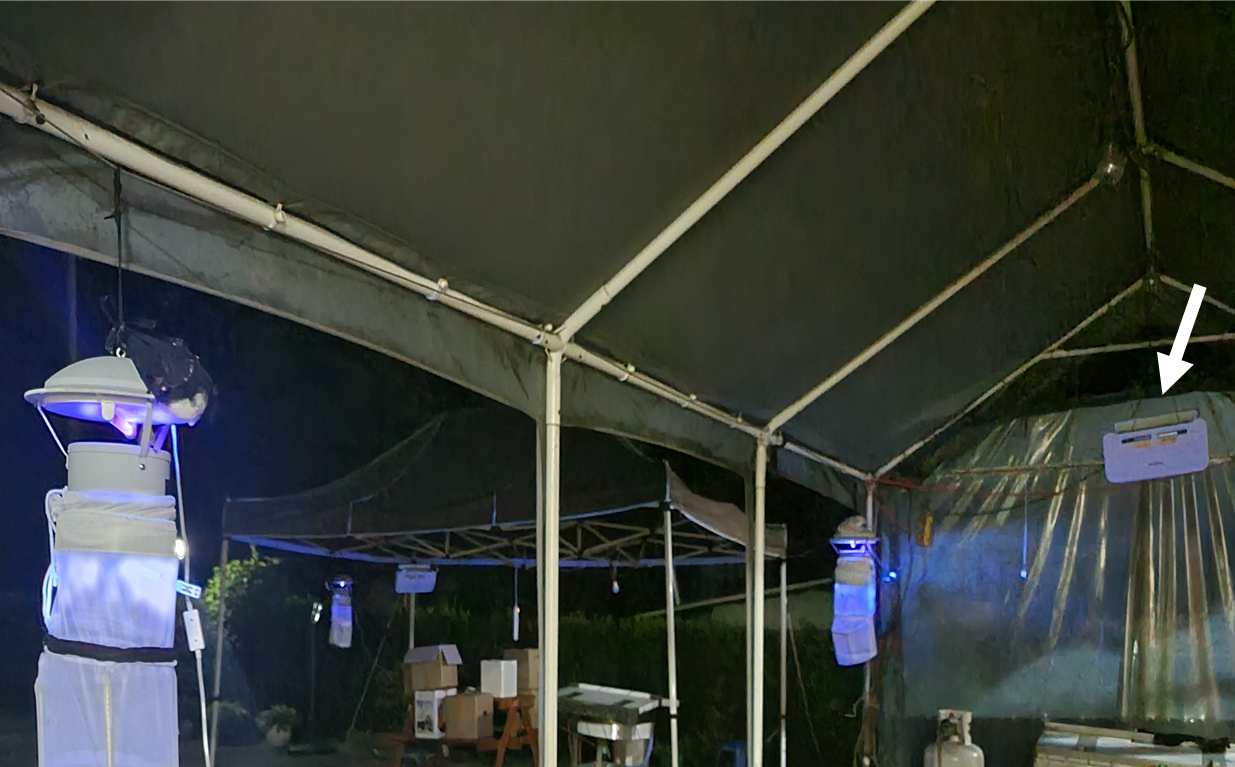
**

**Fig. S9. Electronic mosquitoes trap (arrow) installed by host in human dwelling in Wolgot-myeon.**
